# Metabolic Signatures of Chikungunya Versus Dengue: A Comparative Study

**DOI:** 10.3390/v18080913

**Published:** 2026-08-20

**Authors:** Yuqiu Liu, Meiyi Li, Huili Chen, Xi Liu

**Affiliations:** Department of Infectious Diseases, The Fifth Affiliated Hospital of Sun Yat-sen, Zhuhai 519000, China; liuyq298@mail2.sysu.edu.cn (Y.L.); limy67@mail2.sysu.edu.cn (M.L.); chhuil@mail.sysu.edu.cn (H.C.)

**Keywords:** chikungunya fever, dengue fever, metabolite

## Abstract

Background: Chikungunya virus and dengue virus rank among the most clinically significant mosquito-borne pathogens worldwide, imposing substantial disease burdens in endemic regions. Despite their overlapping symptomatology, the diseases diverge markedly in prognosis. This disparity underscores the critical need for tools to differentiate these conditions and elucidate their distinct pathogenic mechanisms. Method: We conducted liquid chromatography–tandem mass spectrometry-based comparative metabolomic analyses to systematically compare plasma profiles from infected individuals. Results: We collected plasma samples from 26 chikungunya fever (CHIKF) patients, 49 dengue fever (DF) patients and 28 healthy individuals. Clinically, CHIKF patients exhibited a higher prevalence of rash (96.3% vs. 36.7%) and involvement of small joints, while DF patients showed prolonged fever and more severe hematological abnormalities. Metabolically, significant differences were observed in specific metabolites, such as eugenol sulfate, lysophosphatidylcholine (14:0/0:0), and piperine, which were dysregulated in both groups compared to healthy controls. A Kyoto Encyclopedia of Genes and Genomes (KEGG) pathway analysis highlighted unique metabolic perturbations in CHIKF, such as peroxisome proliferator-activated receptor (PPAR) signaling and mechanistic target of rapamycin complex (mTOR) pathways, suggesting potential mechanisms underlying CHIKV-induced arthritis. Conclusions: This study identifies distinct metabolic signatures and functional pathways associated with CHIKF and DF, offering a potential mechanism for differential diagnosis and therapeutic targets. The role of eugenol sulfate in modulating CHIKV-induced arthralgia is particularly promising, supported by its known anti-inflammatory properties.

## 1. Introduction

Arthropod-borne viruses pose an increasing threat to global public health, particularly in tropical and subtropical regions with widespread populations of susceptible mosquito vectors [1]. Among these, both chikungunya virus (CHIKV, Genus *Alphavirus*, *togaviridae*) and dengue virus (DENV, *Flaviviridae* family, genus *Flavivirus*) represent two pathogenic mosquito-borne pathogens that causes the greatest disease burden [2]. CHIKV, a single-stranded positive-sense RNA virus belonging to the *Togaviridae* family (genus *Alphavirus*), is primarily transmitted by *Aedes aegypti* and *Aedes albopictus* mosquitoes [1]. CHIKV infection causes an acute self-limiting disease known as chikungunya fever (CHIKF), characterized by sudden high fever, polyarthritis, and rash [1]. Similarly, DENV, a single-stranded positive-sense RNA virus of the *Flaviviridae* family (genus *Flavivirus*), is also transmitted by *Aedes aegypti* and *Aedes albopictus* [2]. DENV infection causes dengue fever (DF), which can range from a mild febrile illness to severe dengue hemorrhagic fever/dengue shock syndrome, characterized by high fever, thrombocytopenia, plasma leakage, and hemorrhagic manifestations [2].

Clinically, CHIKF and DF exhibit substantial overlap in their manifestations, with shared symptoms including acute fever, rash, and polyarthralgia. This clinical similarity, combined with overlapping geographic distributions, creates significant challenges for differential diagnosis [3]. However, their disease outcomes differ markedly. DF carries a higher risk of progression to life-threatening hemorrhagic fever [4]. In DF patients, joint pain in small joints and large joints is relatively common, but typical arthritis is quite rare [5], while CHIKF more frequently leads to persistent arthropathy. The intense acute joint pain usually affects small joints accompanied by swelling and stiffness, which might persist for months or even years, or develop into chronic arthritis, severely impairing daily function and quality of life [6].

Research on CHIKF-related arthropathy suggests that joint pain may result from damage-associated molecular patterns (DAMPs) released during CHIKV infection. These DAMPs trigger a cytokine storm characterized by elevated levels of pro-inflammatory cytokines and chemokines, including IL-6, IL-17, IL-1β, TNF-α, CXCL8 and CCL2 [7]. The subsequent recruitment of immune cells (e.g., neutrophils and macrophages) exacerbates local inflammation, culminating in acute joint pain. Current treatment strategies for CHIKV-induced arthritis remain palliative, relying on non-steroidal anti-inflammatory drugs (NSAIDs), corticosteroids, analgesics (e.g., acetaminophen, opioids), and neuroactive agents (e.g., gabapentin, amitriptyline) [8]. However, the efficacy of these therapies is often limited, and prolonged use may lead to adverse effects, highlighting the need for more targeted treatment approaches.

Metabolomics, which involves systematic analysis of low-molecular-weight metabolites, provides a powerful tool for monitoring real-time pathophysiological changes during infections [9]. Recent LC-MS/MS-based metabolomic studies have characterized metabolic disturbances in DENV-infected patients. Voge et al. identified serum small-molecule biomarkers associated with distinct DENV infection outcomes in pediatric patients from Nicaragua and Mexico [9]. Cui et al. reported serum metabolome and lipidome alterations in adult patients with primary dengue infection, highlighting disruptions in lipid metabolism and amino acid pathways [10]. More recently, Anshad et al. revealed distinct circulating bioactive lipid signatures in DENV-infected patients [11], while Liu et al. demonstrated ABC transporter disturbances through integrated metabolomics and transcriptomics [12]. These studies collectively identified metabolic pathways involved in viral replication, immune evasion, and disease progression.

In contrast, metabolomic studies on CHIKV infection remain limited. Shrinet et al. performed serum metabolomics analysis of patients with chikungunya and dengue mono/co-infections, revealing distinct metabolite signatures across disease conditions [13]. Byers et al. provided a comprehensive review of metabolomic insights into human arboviral infections, including CHIKV [14]. Nevertheless, no comprehensive studies have directly compared the metabolic profiles of CHIKV and DENV infections using a unified analytical approach, underscoring the need for the present study.

Therefore, this study employs LC-MS/MS-based comparative metabolomics not only to identify shared mechanisms underlying the similar clinical presentations of these viruses but also to establish a foundation for developing novel therapeutic targets specific to CHIKF-associated arthralgia.

## 2. Materials and Methods

### 2.1. Study Design and Data Collection

Our study was approved by the Research Ethics Committee at the Fifth Affiliated Hospital, Sun Yat-sen University. This retrospective observational study was conducted at the Fifth Affiliated Hospital, Sun Yat-sen University, from December 2023 to August 2025. Although this study was designed retrospectively, the plasma sample collection, storage, and processing procedures followed standardized protocols established at the Fifth Affiliated Hospital, Sun Yat-sen University. Diagnostic criteria were applied retrospectively to classify patients, but all samples were collected and processed uniformly at the time of clinical presentation. The study collected plasma samples from 26 CHIKF patients, 49 DF patients and 28 healthy individuals, and informed consent was obtained for all individuals.

Clinical and laboratory data were retrieved retrospectively from the electronic medical records (EMR) of the Fifth Affiliated Hospital, Sun Yat-sen University, China. Demographic information (age, sex), comorbidity history (hypertension, diabetes, coronary heart disease), clinical symptoms (fever duration, maximum body temperature, rash, joint involvement), and laboratory test results (complete blood count, coagulation profile, liver and renal function, inflammatory markers) were extracted using a standardized data collection form. Hematological and biochemical parameters were measured using the hospital’s routine clinical laboratory assays during the patients’ acute-phase hospitalization. Joint involvement was assessed by the attending infectious disease physicians during clinical examination, with affected joints recorded at the time of enrollment.

The inclusion criteria included the following: 1. in the CHIKF group, the epidemiological history and clinical manifestations met the diagnostic criteria for CHIKF [15], which are CHIKF PCR or IgM positive from laboratory confirmation, individuals aged 16 or above, and disease duration (from the first time showing typical CHIKF infection symptoms to the time of enrollment) ≤ 7 days; 2. in the DF group, the epidemiological history and clinical manifestations met the diagnostic criteria for DF [16], which are DF PCR or IgM positive from laboratory confirmation, individuals aged 16 or above, and disease duration (from the first time showing typical DF infection symptoms to the time of enrollment) ≤ 7 days; 3. In the healthy group, indicators such as blood lipids, blood sugar, and liver and kidney functions retained a normal level, there were no abnormalities in electrocardiograms, and there was no critical illness or history of related diseases such as dyslipidemia, diabetes, and cardiovascular diseases in the past. The exclusion criteria included: pregnant or lactating women, those complicated with other arbovirus diseases or systemic viral infections, and patients with congenital or acquired immune deficiencies.

### 2.2. Clinical Sample Collection

The collection of plasma samples was performed as follows. All individuals provided 5 mL of venous blood samples at an empty stomach state (more than 8 h after a meal). At a regular temperature, the blood was collected from a blood collection tube containing EDTA and then gently inverted and mixed the blood immediately. Blood samples were centrifuged at 3500 r/min (radius 17.8 cm) at 4 °C for 10 min. The supernatant was collected and placed in a centrifuge tube and then frozen at −80 °C for testing. Then, the plasma samples stored at −80 °C (Thermo Fisher Scientific, Waltham, MA, USA) were taken out to be thawed in an ice–water mixture. Each 100 μL of plasma was added to 400 μL of the protein precipitation agent methanol–acetonitrile (V:V = 2:1; Fisher Scientific, Waltham, MA, USA, containing a mixture of six internal standards [L-2-chlorophenylalanine, succinic acid-d4, L-valine-d8, cholic acid-d4, D-luciferin, and L-carnitine-d3; Shanghai Yuanye Biochemical Technology Co., Ltd., Shanghai, China; Shanghai Haoyuan Biochemical Technology Co., Ltd., Shanghai, China; Sigma-Aldrich (Merck), St. Louis, MO, USA] for retention time alignment and data normalization) and then vortexed for 1 min (TYXH-I, Shanghai Hannuo Instrument Co., Ltd., Shanghai, China). After that, ultrasonic extraction was performed in an ice water bath (F-060SD, Shenzhen Fuyang Technology Co., Ltd., Shenzhen, China) for 10 min. The samples were stored at −40 °C (Thermo Fisher Scientific, Waltham, MA, USA) overnight before centrifugation (5430R, Eppendorf, Hamburg, Germany) for 20 min (12,000 rpm, 4 °C). At last, 150 μL of the supernatant was transferred to an LC-MS injection vial that had a footed inner liner within for analysis.

### 2.3. Chromatography and Mass Spectrometry Analysis

The samples collected from plasma were pre-segregated using an ACQUITY UPLC HSS T3 column (100 mm × 2.1 mm, 1.8 μm; Waters Corporation, Milford, MA, USA). Mobile phase A was a water solution containing 0.1% formic acid, and mobile phase B was an acetonitrile solution containing 0.1% formic acid (both Fisher Scientific, Waltham, MA, USA). The elution gradient was: (0–2 min, 5% B; 2–4 min, 5–30% B; 4–8 min, 30–50% B; 8–10 min, 50–80%; 10–14 min, 30–100%; 14–15 min, 100%; 15–15.1 min, 100–5%; 15.1–16 min, 5%). Mass spectrometry analysis was then performed using data acquisition on a Thermo QE HF mass spectrometer (Thermo Fisher Scientific, San Jose, CA, USA). The ion source was a high-energy electrospray ionization source (HESI source), and data was collected simultaneously in both positive and negative ion modes as Full-MS/dd-MS2. Ion source parameters were set as follows: in positive ion mode, the spray voltage was 3800 V, and in negative ion mode it was −3200 V. The capillary temperature was set at 320 °C, while the ion source auxiliary temperature was 350 °C. Oher settings included: a sheath gas (N2) flow rate of 35 L/h, an auxiliary gas (N2) flow rate of 8 L/h, a scanning range of m/z 70–1050, and a resolution of 60,000. To ensure data quality and analytical reproducibility, quality control (QC) samples were prepared by pooling equal volumes (10 μL) from each study plasma sample. QC samples were processed identically to study samples and injected at regular intervals (every 10 study samples) throughout the analytical run to monitor system stability. Additionally, solvent blank injections were performed at the beginning and end of each batch to assess background contamination and carryover. This untargeted metabolomic profiling approach aimed to comprehensively detect and quantify small-molecule metabolites (m/z 70–1050) in plasma, encompassing lipids, amino acids, organic acids, nucleotides, and other intermediary metabolites.

### 2.4. Data Analysis

For participants’ characteristics, the testing methods included an independent sample *t*-test, a χ^2^ test and a Mann–Whitney U test. Continuous variables with normal distribution were calculated by an independent sample *t*-test. Other continuous variables used the Mann–Whitney U test. Bivariate was calculated by the χ^2^ test. For metabolite analysis, packages such as ropls (version [3.0]) and geelm (version [4.0]) were used in R (version [4.2.1]; R Core Team) for analysis, and the data was visualized using the ggplot (version [4.0.3]) package (Supplementary Information R package). After data filtering, normalization, and transformation, principal component analysis (PCA) and orthogonal partial least squares discriminant analysis (OPLS-DA) were employed to conduct univariate and multivariate statistical analyses of the data. To screen for differential metabolites, we set the screening criteria as follows: 1. FC > 1.5 or < 0.66; 2. *t*-test *p*-value < 0.05; 3. VIP > 1; 4. level: Level 1 or Level 2. After the differential metabolites were obtained, the pathways of the differential metabolites were further analyzed using the Kyoto Encyclopedia of Genes and Genomes (KEGG) database to identify the metabolic pathways that underwent significant changes. The four-tier metabolite identification confidence levels were assigned according to the Metabolomics Standards Initiative (MSI) guidelines: Level 1 (identified compounds, confirmed by MS/MS spectral matching against authentic standards and retention time alignment) and Level 2 (putatively annotated compounds, matched by MS/MS spectral similarity to public databases including HMDB, MassBank, and LipidMaps). Only metabolites reaching Level 1 or 2 were retained for downstream pathway analysis to ensure annotation reliability. Pathway enrichment was performed on the filtered differential metabolites using the KEGG database, with pathway significance determined by a hypergeometric test (*p* < 0.05) and an impact factor > 0.

Regarding the single-sample-per-patient design, this study employed a cross-sectional comparison framework rather than longitudinal tracking. Metabolic pathway alterations were inferred from between-group differences (CHIKF vs. DF, CHIKF vs. healthy, DF vs. healthy) at the acute phase, not from within-individual temporal changes. Each group comprised independently sampled patients at comparable acute-phase time points (≤7 days from symptom onset), and group-level metabolic pathway enrichment reflects the aggregate perturbation profile characteristic of each disease state. While this design cannot capture intra-individual metabolic trajectory over time, it is well-suited for identifying disease-specific metabolic signatures through cross-sectional group comparisons.

At last we selected metabolites using the index words “metabolite’s name + “Infections”[Mesh] OR (“Infection”[Title/Abstract]) OR (“Infestation”[Title/Abstract]) OR (“Infection and Infestation”[Title/Abstract]) OR (“Infestation and Infection”[Title/Abstract]) OR (“Infections and Infestations”[Title/Abstract]) OR (“Infestations and Infections”[Title/Abstract]) infection + “Fever”[Mesh] OR (“Fevers”[Title/Abstract]) OR (“Pyrexia”[Title/Abstract]) OR (“Pyrexias”[Title/Abstract]) + “Arthralgia”[Mesh] OR (“Arthralgias”[Title/Abstract]) OR (“Joint Pain”[Title/Abstract]) OR (“Joint Pains”[Title/Abstract]) OR (“Pain, Joint”[Title/Abstract]) OR (“Pains, Joint”[Title/Abstract]) OR (“Polyarthralgia”[Title/Abstract]) OR (“Polyarthralgias”[Title/Abstract]) + “Exanthema”[Mesh] OR (“Exanthem”[Title/Abstract]) OR (“Rash”[Title/Abstract]) OR (“Skin Rash”[Title/Abstract]) OR (“Rash, Skin”[Title Abstract]) + “Viruses”[Mesh] OR (“Virus”[Title/Abstract]) OR (“Animal Viruses”[Title/Abstract]) OR (“Animal Virus”[Title/Abstract]) OR (“Virus, Animal”[Title/Abstract]) OR (“Viruses, Animal”[Title/Abstract]) OR (“Zoophaginae”[Title/Abstract])” in Pubmed for further research.

## 3. Results

### 3.1. Participants’ Characteristics

A total of 26 patients with confirmed CHIKF, 49 patients with confirmed DF and 28 healthy individuals were included (Table 1, Supplementary Information Table S1). The mean age of the CHIKF patients (43.38 ± 11.89 years) did not significantly differ from that of DF patients (42.90 ± 16.17 years; *p* = 0.883). However, the gender distribution differed between the two groups: CHIKF patients were predominantly male (76.9%), whereas the DF group exhibited a more balanced gender ratio (46% male, 54% female). Comorbidities such as hypertension, diabetes, and coronary heart disease were rare in both groups. Specifically, CHIKF patients included two cases of hypertension, one case of diabetes, and none with coronary heart disease, while the DF group had six cases of hypertension, six of diabetes, and two of coronary heart disease.

The primary symptoms in CHIKF patients included fever, rash, and joint pain. Notably, the duration of fever was significantly longer in DF patients (5.41 ± 2.05 days) compared to CHIKF patients (2.59 ± 1.90 days; *p* < 0.000). Although DF patients exhibited a higher mean body temperature (39.02 ± 0.67 °C) than CHIKF patients (36.54 ± 9.15 °C), this difference was not statistically significant (*p* = 0.266). Rash was present in nearly all CHIKF patients (96.3%), compared to only 36.7% of DF patients (*p* < 0.000). Joint pain also differed between the groups: CHIKF patients experienced an average of 2.63 large joints and 2.54 small joints affected, while DF patients showed 4.33 large joints affected and no small joint involvement.

Laboratory analyses revealed significant differences in several hematological and biochemical parameters. DF patients had significantly lower counts of white blood cells (*p* = 0.000), neutrophils (*p* = 0.000), lymphocytes (*p* = 0.008), eosinophils (*p* = 0.000), and platelets (*p* = 0.000), as well as lower creatine kinase levels (*p* = 0.031). Conversely, DF patients exhibited higher levels of aspartate aminotransferase (*p* = 0.000), the estimated glomerular filtration rate (*p* = 0.018), activated partial thromboplastin time (*p* = 0.000), and D-dimer (*p* = 0.000), indicative of more severe infection and hemorrhage. No other significant differences were observed (Table 1).

### 3.2. Distribution of Joint Pain

**Figure 1:**
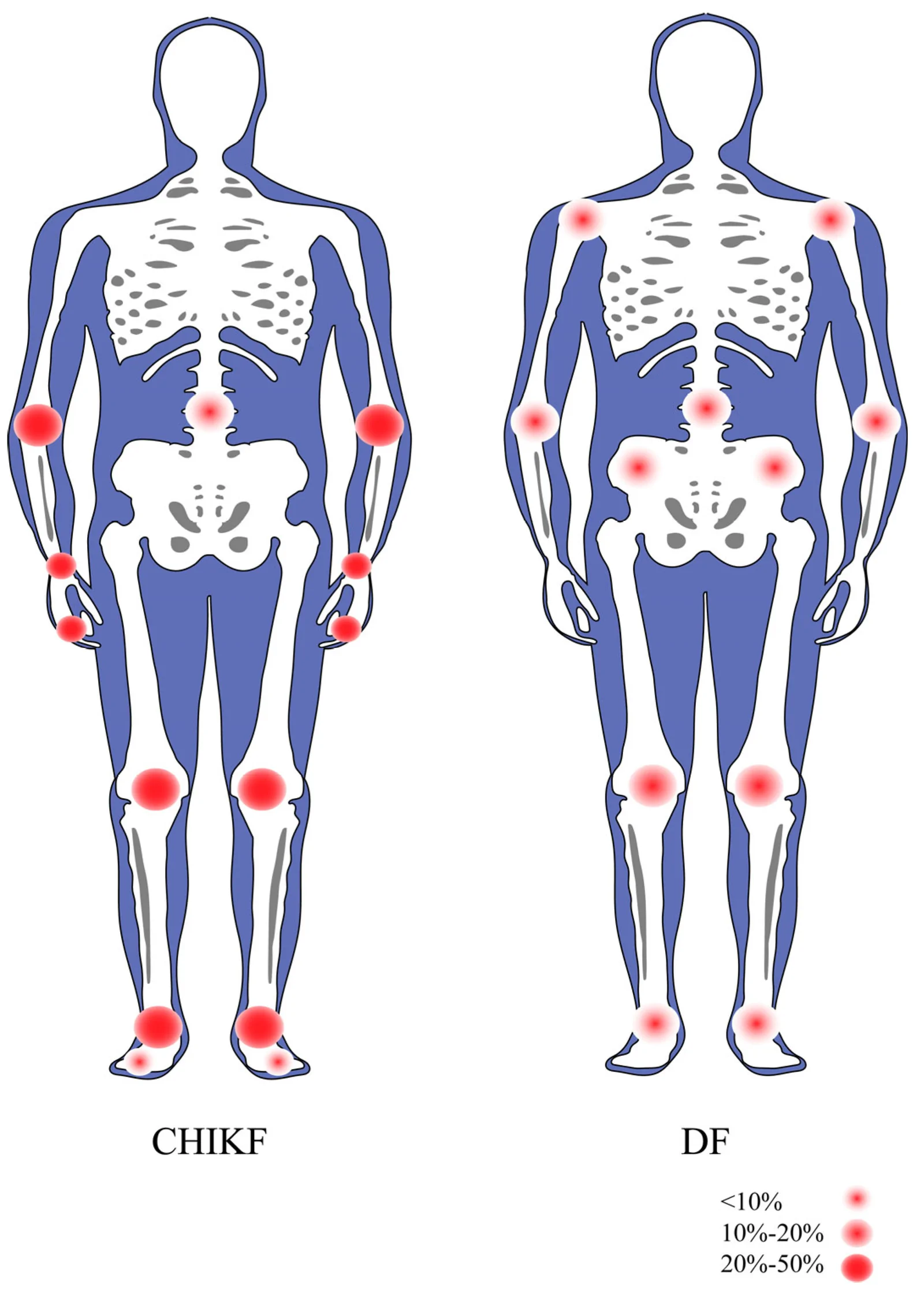
Distribution of joint pain. Schematic diagram of the distribution of joint pain.

The distribution of joint pain is illustrated in Table 2 and Figure 1. Among CHIKF patients, large joints most affected included the knee (42.31%), elbow (23.09%), ankle (26.92%), lumbar vertebrae (3.85%), and limbs (3.85%). In contrast, DF patients primarily exhibited involvement of central large joints (10.20% knee, 4.08% elbow, 4.08% ankle, 2.04% lumbar vertebrae, 2.04% hip, and 6.12% shoulder). Notably, small joint involvement was exclusive to CHIKF patients, affecting the wrist joint (34.62%), finger joints (26.92%), and toe joints (3.85%).

### 3.3. Metabolite Profiling

**Figure 2:**
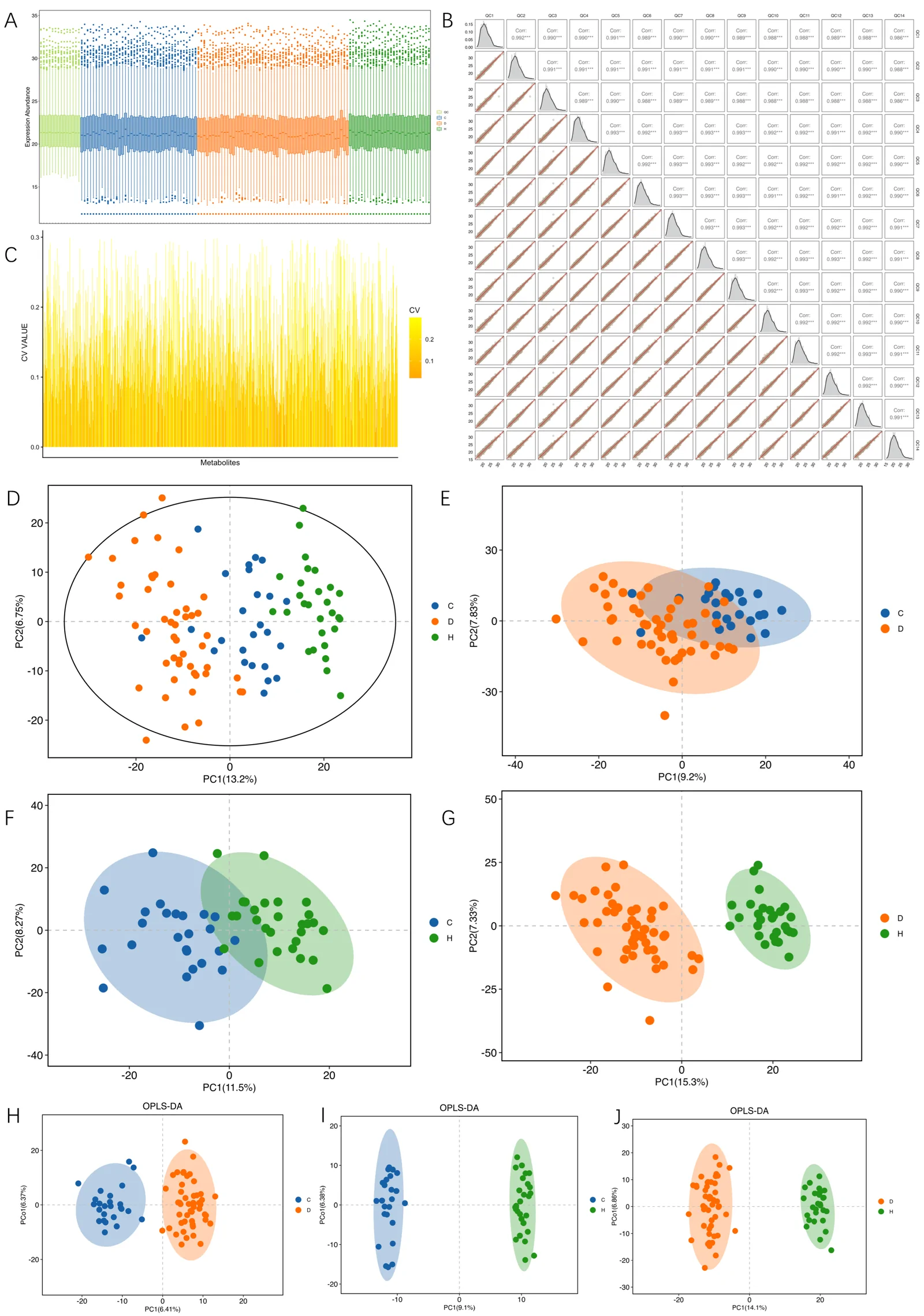
Quality analysis and plasma metabolomic analysis of CHIKF, DF and healthy groups. (**A**) Box plot of sample metabolite intensities for 1244 metabolites. (**B**) Correlation among QC samples for 1244 metabolites. (**C**) Coefficient of variation (CV) values for 1244 metabolites. (**D**) PCA scatter plot displaying the distribution of samples across baseline CHIKF group (C), DF group (D), and healthy group (H) on the first two principal components. (**E**) PCA scatter plot displaying the distribution of samples across CHIKF group and DF group. (**F**) PCA scatter plot displaying the distribution of samples across CHIKF group and healthy group. (**G**) PCA scatter plot displaying the distribution of samples across DF group and healthy group. (**H**–**J**) OPLS-DA scatter plot displaying the distribution of samples across clinical groups (C vs. D, C vs. H, and D vs. H). Correlations are marked with *** where the correlation coefficient r > 0.99 (*p* < 0.001), indicating a highly significant association.

Non-targeted metabolomic analysis was conducted on plasma samples from 103 participants, quantifying a total of 1244 metabolites. Metabolites with missing values exceeding 20% were set to using half the minimum value observed in the dataset (Supplementary Information Table S2). Quality control (QC) results demonstrated robust assay stability, as evidenced by: (1) uniform median intensities across sample metabolite distributions (Figure 2A), (2) high inter-QC sample correlations (r > 0.9; Figure 2B), and a (3) low coefficient of variation (CV < 0.3; Figure 2C).

Principal component analysis (PCA) of metabolite profiles revealed distinct clustering among the CHIKF, DF, and healthy groups (Figure 2D–G). The separation between DF and healthy controls was particularly pronounced, consistent with the clinical severity of DF. Orthogonal partial least squares discriminant analysis (OPLS-DA) further validated these group distinctions (Figure 2H–J).

**Figure 3:**
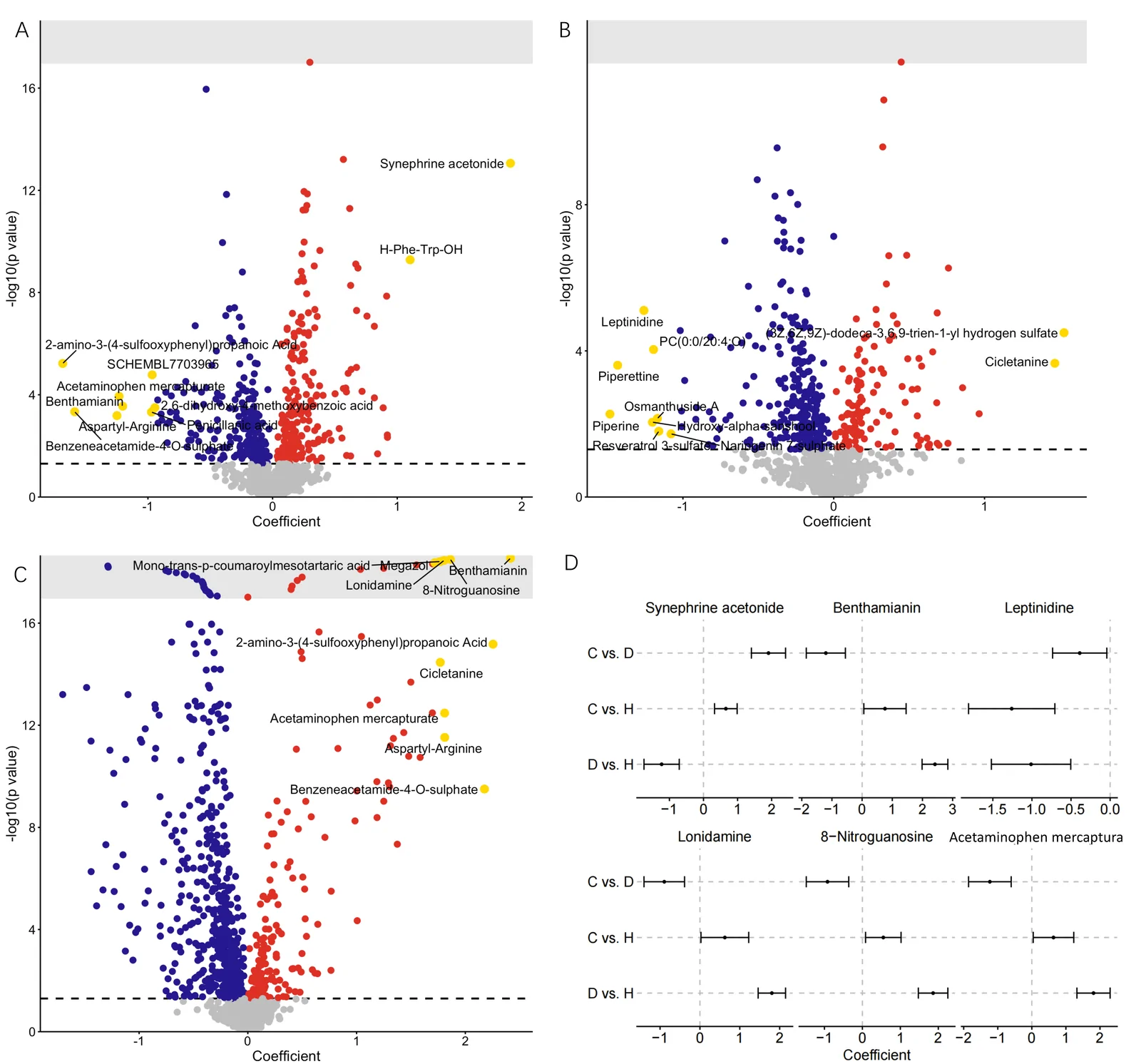
Plasma metabolomic differences among CHIKF, DF and healthy groups. (**A**–**C**) Volcano plot showing metabolite associations across clinical groups (C vs. D, C vs. H, and D vs. H), via generalized estimating equation (GEE) model, adjusted for age, sex, comorbidities and duration of disease. Significance based on coefficient > 0, *p* < 0.05. Metabolites with positive associations are shown in red, negative are shown in blue. The top 10 most significant metabolites are highlighted in yellow. Gray region represents metabolites with *p*-value above the detectable threshold. (D, E, F) Screening of differential metabolites. Screening criteria: 1. FC > 1.5 or < 0.66. 2. *t*-test *p*-value < 0.05. 3. VIP > 1. 4. Level: Level 1, Level 2. (**D**) Common metabolites that differ among all three controls.

To account for confounding clinical variables, generalized estimating equation (GEE) models adjusted for age, sex, comorbidities, and disease duration were employed. Differentially abundant metabolites were filtered using the following criteria: (1). fold change (FC) > 1.5 or <0.66; (2). significance (*p* < 0.05) in GEE-derived *t*-tests; (3). metabolite confidence level: Tier 1 or 2; (4). variable importance in projection (VIP) > 1. For the CHIKF vs. DF comparison, 120 metabolites were identified (70 upregulated, 50 downregulated). The CHIKF vs. healthy comparison yielded 120 metabolites (25 upregulated, 95 downregulated), while the comparison of DF vs. healthy individuals revealed 170 metabolites (51 upregulated, 119 downregulated; Figure 3A–D). Top-ranked metabolites based on FC magnitude are summarized in Table 3A–C.

### 3.4. KEGG Analysis and Metabolites Screening

**Figure 4:**
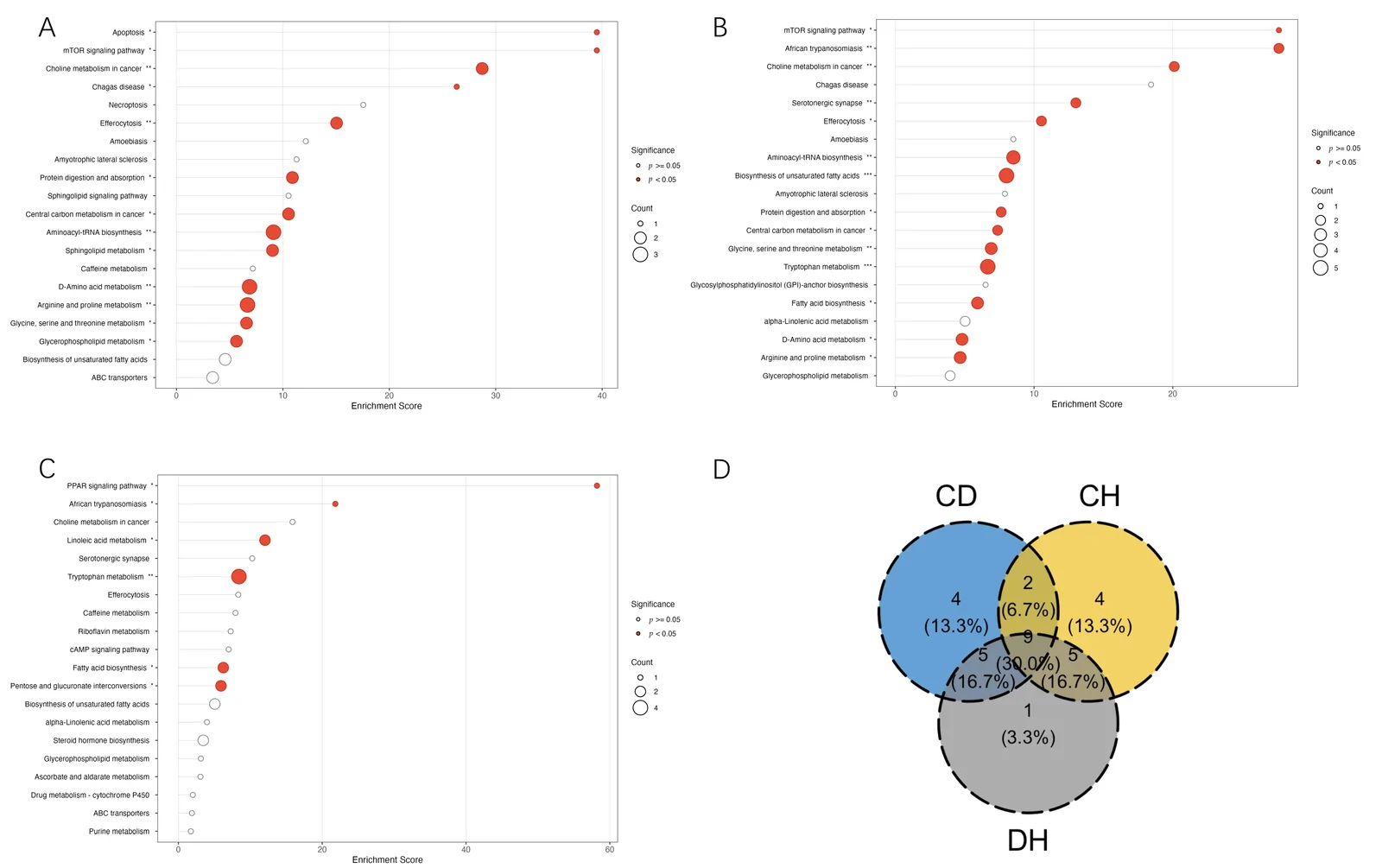
KEGG pathway analysis and metabolite screening. (**A**–**C**) KEGG pathway analysis. A: CHIKF vs. DF, based on 120 metabolites. B: CHIKF vs. healthy group, based on 120 metabolites. C: DF vs. healthy group, based on 170 metabolites. (**D**) Venn plot of KEGG pathway between CHIKF, DF and healthy groups. CHIKF vs. DF (CD), CHIKF vs. healthy (CH), DF vs. healthy (DH).

To elucidate potential disease mechanisms, we performed KEGG enrichment analysis on the filtered metabolites (Figure 4A–D). Key findings included the following: in the CHIKF vs. DF: comparison, enriched pathways included choline metabolism in cancer and African trypanosomiasis (Figure 4A). In the CHIKF vs. healthy comparison, significant pathways involved PPAR signaling and mTOR signaling (Figure 4B). In the DF vs. healthy comparison, enriched pathways comprised linoleic acid metabolism, African trypanosomiasis, and efferocytosis (Figure 4C).

The Venn diagram (Figure 4D) highlights shared pathways across comparisons (e.g., arginine biosynthesis, glycine/serine metabolism), suggesting conserved host responses. Notably, nine pathways were common to both the CHIKF vs. healthy and DF vs. healthy comparisons, while five pathways (e.g., PPAR signaling, purine metabolism) were unique to CHIKF (Supplementary Information Table S3).

### 3.5. Potential Mechanisms of Arthritis in CHIKF

Metabolite trends in CHIKF and DF patients exhibited a convergent pattern: metabolites elevated in CHIKF were further upregulated in DF, while those suppressed in CHIKF were more profoundly downregulated in DF (Supplementary Information Table S4). Candidate metabolites associated with arthritis pathogenesis included the following: lysophosphatidylcholine (LysoPC) (14:0/0:0) and isophorone were downregulated and piperine, oleoylethanolamide (OEA), and phthalic acid were upregulated.

Of particular interest was eugenol sulfate, a metabolite showing altered levels between CHIKF and DF, though it did not meet all the differential screening criteria (*p* = 0.27, Level 4). To evaluate whether candidate metabolites were associated with arthritis severity, Spearman correlation, multiple linear regression (adjusted for sex and age), and Kruskal–Wallis tests were performed for all 13 candidate metabolites against large and small joint counts. No significant associations were observed across any metabolite or method (all *p* > 0.05; Supplementary Information Figures S1 and S2; Table S5), suggesting that these plasma metabolites may reflect systemic perturbations rather than joint-specific biochemical changes.

## 4. Discussion

This study employed LC-MS/MS-based metabolomics to compare plasma profiles of CHIKF and DF patients, revealing distinct metabolic signatures between these clinically similar yet pathologically distinct mosquito-borne diseases (Figure 4). Several key metabolites showed significant alterations, including LysoPC (14:0/0:0), piperine, oleoylethanolamide (OEA), phthalic acid, and eugenol sulfate—each offering unique insights into disease mechanisms and therapeutic potential.

LysoPC (14:0/0:0): The significant downregulation of LysoPC (14:0/0:0) in both CHIKF and DF patients (*p* < 0.05) is associated with viral pathogenesis. While this phospholipid derivative has demonstrated anti-inflammatory effects in LPS-induced models by suppressing IL-1β, IL-6, and TNF-α [15], its depletion in our study suggests a possible pro-inflammatory mechanism during arboviral infections. This paradoxical observation may reflect tissue-specific effects, as LysoPC has shown opposing correlations in cancer progression [17]. Further research should clarify whether LysoPC supplementation could ameliorate viral arthritis.

Piperine: The upregulation of piperine in infected groups (*p* < 0.05) is consistent with reports of potential antiviral properties. Recent in silico studies indicate piperine may disrupt CHIKV entry by binding E1-E2 glycoproteins [18], while its superior bioavailability compared to conventional antivirals [19] suggests potential therapeutic relevance. Our findings suggest that piperine may warrant further investigation as a potential biomarker, as it showed a significant upregulation in both disease groups.

Oleoylethanolamide (OEA): Elevated OEA levels in patients correlate with its known PPAR-α-mediated anti-inflammatory effects [20]. Particularly relevant is OEA’s demonstrated ability to inhibit synovial hyperplasia in rheumatoid arthritis models [21], which may be associated with joint-related inflammatory processes. The shared elevation in DF and CHIKF may reflect a shared host response against Flavivirus and Alphavirus infections.

Phthalic acid: Plasma metabolomics also revealed that compared to the healthy group, phthalic acid could transform to common metabolite as phthalic acid [22]. While some phthalate derivatives show antiviral activity [23], their insecticidal properties against *Aedes vectors* suggest our findings may reflect either host defense responses or environmental exposure histories [24].

Notably, acetaminophen metabolites were profoundly elevated (∼100×) in patients, consistent with its widespread use for fever management [25]. There were five patients with CHIKF and 17 patients with DF that had used acetaminophen (Supplementary Information Table S1). This underscores the need to distinguish drug-derived metabolic changes from disease signatures in future studies.

Research showed that eugenol had function of antiinflammation, analgesic and antioxidative effect [26]. While eugenol sulfate did not meet all differential screening thresholds in our cohort, we were particularly interested in its potential relevance to CHIKV-induced arthritis given its parent compound’s known anti-inflammatory, analgesic, and antioxidative effects [12]. Eugenol exerts these effects through inhibiting nuclear factor-κB activation by lipopolysaccharide, suppressing cytokine release and macrophage cyclooxygenase-2 expression, and inhibiting 5-lipoxygenase activity in polymorphonuclear cells [27]. In animal trials, eugenol has been reported to alleviate joint pain in mice [28,29]. More importantly, benzoxazole derivatives of eugenol have been reported to interfere with CHIKV replication, demonstrating potent and selective antiviral properties that outperform ribavirin and aciclovir [25]. However, as our metabolomics data did not show significant differences for eugenol sulfate and joint-pain correlations were non-significant across all 13 candidate metabolites, these observations should be considered hypothesis-generating rather than confirmatory [30]. It should be noted that CHIKF-associated polyarthritis manifests during the acute phase of infection, typically within days of symptom onset, and was therefore assessable within our ≤7-day enrollment window. The joint involvement recorded in our cohort (Table 1) reflects this acute arthritic presentation rather than chronic arthropathy.

Regarding sample collection timing, the median disease duration at blood sampling was 0 days (range 0–5) for CHIKF patients and 2 days (range 0–7) for DF patients, both within the 7-day acute-phase window specified by our inclusion criteria. Notably, both groups were sampled predominantly within the early acute phase, with CHIKF patients sampled at a median of day 0 (i.e., on the day of symptom onset or before symptom recognition) and DF patients at a median of day 2 (Table S5). Although metabolomic profiles may shift dynamically during acute infection, two factors mitigate the timing-related concern. First, the GEE models used for differential metabolite identification were adjusted for disease duration alongside age, sex, and comorbidities, thereby accounting for potential timing-related confounding. Second, the relatively narrow sampling window and early acute-phase collection in both groups (median ≤ 2 days) reduce the likelihood that inter-group metabolic differences were driven by sampling timing rather than disease-specific perturbations. The shorter sampling interval in CHIKF patients (median 0 days vs. 2 days in DF) may reflect the more abrupt clinical presentation of CHIKF, which typically prompts earlier medical attention. Nonetheless, we cannot fully exclude the possibility that intra-group temporal variation contributed to residual noise, and future studies with serial sampling across multiple time points would help characterize the trajectory of metabolic changes during acute CHIKV and DENV infection.

To validate the consistency of our metabolomic findings with previous DENV studies, we compared our DF vs. healthy control results with two independent cohorts reported by Cui et al. (Singapore) [10] and Voge et al. (Nicaragua/Mexico) [9]. Our enrichment of tryptophan metabolism, arginine biosynthesis, glycine–serine–threonine metabolism, and citrate cycle aligns with Cui et al.’s report of altered tryptophan, amino acid, and energy metabolism pathways in adult dengue patients [10]. Our observed disturbances in multiple LysoPC species and unsaturated fatty acid biosynthesis are consistent with their lipid-focused findings and with Voge et al.’s identification of LysoPC (14:0/16:0/18:1) perturbations in DENV-infected patients [9]. Notably, the additional enrichment of efferocytosis and African trypanosomiasis pathways in our cohort may reflect population-specific or methodological differences and warrants further investigation. The convergence of our DF findings with prior studies across geographically distinct populations supports the robustness of our analytical approach.

An important consideration is that plasma metabolomics reflects systemic metabolic perturbations rather than tissue-specific changes at sites of viral replication or inflammation. CHIKV primarily replicates in joint synovial macrophages, fibroblasts, and muscle cells, and the metabolic milieu at these local tissue sites may differ substantially from the circulating plasma metabolome. Consequently, the metabolic signatures identified in this study should be interpreted as systemic host responses to infection rather than direct reflections of local joint biochemistry. For instance, the absence of significant correlations between candidate metabolites and joint-pain severity may partly reflect this systemic–local disconnect, as plasma levels of metabolites such as eugenol sulfate may not accurately represent their concentrations or activities at the site of arthritic inflammation. Future studies employing synovial fluid metabolomics or tissue-level metabolomic profiling would be valuable for capturing joint-specific metabolic alterations and for bridging the gap between systemic biomarkers and local pathogenic processes.

However, our research had several limits: (1) the relatively small sample size limited statistical power, particularly for detecting modest effect sizes; (2) plasma metabolomics reflects systemic metabolism and may not accurately capture joint-specific biochemical changes; (3) joint-pain correlation analyses did not reach significance for any of the 13 candidate metabolites, precluding direct mechanistic conclusions about arthritis severity; (4) functional validation through in vitro or in vivo studies is required to confirm the hypothesized roles of these metabolites; (5) medication use (e.g., acetaminophen) may have introduced confounding effects on metabolite levels; (6) the ≤7-day sampling window captured only the acute-phase metabolic profile, and our findings therefore reflect acute host responses rather than predictors of disease progression. In particular, these cross-sectional acute-phase metabolites cannot be used to predict which CHIKF patients will develop chronic arthritis or which DF patients will progress to DHF/DSS. Longitudinal studies with serial sampling across acute, convalescent, and chronic phases are needed to identify prognostic biomarkers for disease progression.

## 5. Conclusions

This study employed LC-MS/MS-based metabolomics to elucidate the distinct pathological mechanisms underlying CHIKF and DF. Through comprehensive KEGG pathway analysis and literature correlation, we identified six key metabolites—LysoPC (14:0/0:0), piperine, acetaminophen mercapturate, 3,4-Dihydroxybenzaldehyde, oleoylethanolamide, and phthalic acid—that demonstrate significant involvement in disease pathogenesis. Particularly noteworthy is eugenol sulfate, which, while not reaching statistical significance in our differential screening criteria, warrants further investigation given its parent compound’s established anti-inflammatory properties and the anti-CHIKV activity of its benzoxazole derivatives.

Several limitations should be acknowledged, including the relatively small sample size and the need for functional validation of these metabolic findings. Nevertheless, our results provide valuable insights into both shared and distinct metabolic pathways between these clinically similar arboviral infections. The identified metabolites not only advance our understanding of disease mechanisms but also offer potential biomarkers for differential diagnosis and therapeutic targets, particularly for CHIKF-associated arthritis. Future studies with larger cohorts and experimental validation are warranted to translate these findings into clinical applications.

## Figures and Tables

**Table 1:** Clinical characteristics.

Clinical Characteristics	CHIKF (N = 26)	DF (N = 49)	*p*	Health (*n* = 28)
Basic				
Age, years ^1^	43.38 ± 11.89	42.90 ± 16.17	0.883	29.59 ± 5.15
Male, *n* (%) ^2^	20 (76.9)	23 (46%)	<0.001 *	13 (46.4%)
Hypertention, *n* (%) ^2^	2 (7.7%)	6 (12.2%)	UA	0 (0%)
Diabetes, *n* (%) ^2^	1 (3.8%)	6 (12.2%)	UA	0 (0%)
Coronary heart disease, *n* (%) ^2^	0 (0%)	2 (4.1%)	UA	0 (0%)
Symptom				
Duration of fever, days ^1^	2.59 ± 1.90	5.41 ± 2.05	<0.001 *	UA
Maximum temperature, °C ^1^	36.54 ± 9.15	39.02 ± 0.67	0.266	UA
Rash, *n* (%) ^2^	26 (96.3)	18 (36.7)	<0.001 *	
Large joint affected, *n* ^1^	2.63 ± 1.78	4.33 ± 2.06	0.007 *	UA
Small joint affected, *n* ^1^	2.54 ± 1.80	0	UA	UA
Laboratory index, *n*				
White blood cell	4.83 (3.74, 5.99)	2.92 (2.09, 3.73)	<0.001 *	UA
Neutrophil	3.23 (2.07, 4.22)	1.59 (1.01, 2.19)	<0.001 *	UA
Lymphocyte	1.13 (0.70, 1.45)	0.69 (0.44, 1.00)	0.008 *	UA
Eosinophil	0.07 (0.01, 0.11)	0.01 (0.00, 0.03)	<0.001 *	UA
Red blood cell	4.75 (4.42, 5.06)	4.62 (4.39, 5.00)	0.472	UA
Hemoglobin	145.00 (132.00, 155.00)	137.00 (127.50, 148.25)	0.164	UA
Platelet ^1^	201.65 ± 49.53	134.54 ± 52.96	<0.001 *	UA
Alanine aminotransferase	21.85 (18.75, 34.52)	28.60 (18.40, 44.35)	0.419	UA
Aspartate aminotransferase	25.60 (18.75, 34.52)	39.30 (31.08, 52.65)	<0.001 *	UA
Total bilirubin	10.90 (7.95, 12.45)	9.25 (7.00, 12.55)	0.365	UA
Direct bilirubin	4.05 (3.08, 5.25)	3.30 (2.30, 4.40)	0.065	UA
Albumin	40.75 (37.95, 45.00)	40.05 (37.85, 41.85)	0.216	UA
Urea	4.51 (2.95, 5.11)	3.45 (2.30, 8.26)	0.186	UA
Serum creatinine ^1^	71.23 ± 12.36	76.28 ± 20.85	0.198	UA
eGFR ^1^	104.98 ± 12.38	95.49 ± 21.00	0.018 *	UA
PT,	10.60 (10.30, 11.20)	11.05 (10.60, 11.68)	0.083	UA
INR	0.94 (0.90, 0.97)	0.96 (0.92, 1.02)	0.158	UA
APTT	27.10 (25.30, 29.20)	29.95 (28.08, 32.08)	<0.001 *	UA
D-dimer	0.26 (0.22, 0.34)	0.72 (0.38, 1.34)	<0.001 *	UA
CRP	6.87 (2.26, 12.24)	3.04 (0.94, 11.44)	0.166	UA
CK	104.55 (76.23, 145.08)	149.60 (105.58, 220.88)	0.031 *	UA
CKMB	11.55 (8.45, 15.65)	13.20 (8.75, 17.65)	0.223	UA

Note: Continuous variables are presented as mean values (range) ^1^, and categorical variables are displayed as counts (percentages) ^2^. eGFR: estimated glomerular filtration rate; PT: prothrombin time; INR: international normalized ratio; APTT: activated partial thromboplastin time; CRP: C-reactive protein; CK: creatine kinase; CKMB: creatine kinase-MB isoenzyme; UA: unable to analyze. *p* < 0.01 is marked with *.

**Table 2:** Joint distribution.

Joint	Position	CHIKV (N = 26)	DF (N = 49)
Large joint	Knee	42.31%	10.20%
	Elbow	23.08%	4.08%
	Ankle	26.92%	4.08%
	Lumbar vertebra	3.85%	2.04%
	Hip joint	0.00%	2.04%
	Shoulder joint	0.00%	6.12%
	Large joints of the limbs	3.85%	0.00%
Small joint	Wrist joint	34.62%	0.00%
	Finger joints	26.92%	0.00%
	Toe joint	3.85%	0.00%

**Table 3:** Top 20 differential metabolites.

**A.** The 20 differential metabolites between CHIKF and DF, totaling 120.					
**ID**	**Metabolites**	**KEGG**	**VIP**	**Level**	**|Log2FC|**
FT2145PR	N-[4′-hydroxy-(E)-cinnamoyl]-L-aspartic acid		2.20	Level 2	3.47
FT1299NR	2,3,6,7-Tetrahydro-6,7-dimethylcyclopent[b]azepin-8(1H)-one		2.54	Level 1	3.44
FT1669NR	Serylphenylalanine		2.78	Level 2	3.33
FT0751NR	3-(2-Furanyl)-2-propenal		2.79	Level 1	2.84
FT2210PR	Enigmol		1.58	Level 1	2.79
FT2863NR	Dihydroferulic acid 4-O-glucuronide		2.72	Level 1	2.43
FT1634PR	Annuionone B		2.25	Level 2	2.37
FT3829PR	LysoPC(18:0/0:0)	C04230	2.13	Level 1	2.37
FT0894NR	alpha-Furyl methyl diketone		2.89	Level 1	2.27
FT1107PR	Cotinine		2.31	Level 1	2.26
FT2284PR	Acetaminophen mercapturate		3.22	Level 2	1.94
FT3482PR	LysoPC(14:0/0:0)		1.86	Level 1	1.83
FT0886NR	Penicillanic acid		2.56	Level 2	1.82
FT0497NR	HOHA lactone		2.32	Level 1	1.81
FT3988PR	LysoPG(20:3/0:0)		3.34	Level 2	1.81
FT3670NR	LysoPE(18:4/0:0)		1.27	Level 2	1.75
FT1018PR	Phthalic acid	C01606	2.29	Level 2	1.72
FT3415NR	Dextran-70		1.09	Level 1	1.68
FT4624NR	OG-PC		2.09	Level 2	1.68
FT3783PR	LysoPC(18:3/0:0)		1.74	Level 1	1.62
**B.** The top 20 differential metabolites between CHIKF and the healthy group, totaling 120.					
**ID**	**Metabolites**	**KEGG**	**VIP**	**Level**	**|Log2FC|**
FT3071PR	Leptinidine		3.63	Level 1	4.36
FT2048PR	Piperine	C03882	4.53	Level 1	3.99
FT1107PR	Cotinine		2.53	Level 1	3.97
FT1826PR	Cicletanine		3.13	Level 1	3.29
FT1173NR	3-Methyldioxyindole	C05834	2.26	Level 1	2.81
FT2527NR	Naringenin 7-sulphate		2.66	Level 1	2.67
FT3793NR	10-Hydroxy-octadec-12Z-enoate-9-beta-D-glucuronide		3.62	Level 1	2.38
FT4012PR	PC(0:0/20:4;O)		3.33	Level 1	2.37
FT1668NR	Phenylmethylglycidic ester		3.24	Level 2	2.29
FT3822NR	Pregnanediol 3-O-glucuronide	C03033	1.82	Level 1	2.18
FT0894NR	alpha-Furyl methyl diketone		1.59	Level 1	1.96
FT3376PR	LysoPC(O-14:0/0:0)		2.55	Level 1	1.95
FT1686NR	5-(3’,5’-Dihydroxyphenyl)-gamma-valerolactone		1.42	Level 1	1.95
FT2299NR	5S,8R-DiHODE	C20186	2.35	Level 1	1.90
FT3614PR	Lupinisoflavone N		3.14	Level 2	1.82
FT0896NR	Isophorone	C14743	2.33	Level 2	1.78
FT1133NR	3,10-dihydroxydecanoic acid		2.61	Level 1	1.70
FT0911NR	3-oxo capric acid	C20714	2.54	Level 1	1.70
FT1018PR	Phthalic acid	C01606	1.44	Level 2	1.68
FT2334PR	Sinapoylputrescine		2.28	Level 2	1.65
**C.** The top 20 differential metabolites between DF and the healthy group, totaling 170.					
**ID**	**Metabolites**	**KEGG**	**VIP**	**Level**	**|Log2FC|**
FT2284PR	Acetaminophen mercaptu2.		2.90	Level 2	9.16
FT1371NR	Benzeneacetamide-4-O-sulphate		3.38	Level 1	7.96
FT0886NR	Penicillanic acid		2.36	Level 2	7.82
FT2145PR	N-[4’-hydroxy-(E)-cinnamoyl]-L-aspartic acid		2.60	Level 2	5.40
FT1669NR	Serylphenylalanine		2.56	Level 2	5.22
FT1299NR	2,3,6,7-Tetrahydro-6,7-dimethylcyclopent[b]azepin-8(1H)-one		2.34	Level 1	4.77
FT3793NR	10-Hydroxy-octadec-12Z-enoate-9-beta-D-glucuronide		3.15	Level 1	4.54
FT0894NR	alpha-Furyl methyl diketone		2.84	Level 1	4.34
FT2773PR	Gln-Ile-Met		1.25	Level 2	4.13
FT0848PR	Acetaminophen	C06804	1.81	Level 2	3.83
FT1826PR	Cicletanine		2.92	Level 1	3.78
FT1018PR	Phthalic acid	C01606	2.44	Level 2	3.49
FT1668NR	Phenylmethylglycidic ester		2.83	Level 2	3.48
FT0895NR	3,4-Dihydroxybenzaldehyde	C16700	2.48	Level 2	3.25
FT3376PR	LysoPC(O-14:0/0:0)		2.80	Level 1	3.10
FT2048PR	Piperine	C03882	2.74	Level 1	3.10
FT3071PR	Leptinidine		2.41	Level 1	3.05
FT4012PR	PC(0:0/20:4;O)		2.88	Level 1	3.02
FT1935NR	Leucylphenylalanine		2.38	Level 1	2.98
FT2400PR	Oleoylethanolamide	C20792	2.49	Level 1	2.87

## Data Availability

The datasets generated during and/or analyzed during the current study are available from the corresponding authors upon request.

## Supplementary Materials

The following supporting information can be downloaded at https://www.mdpi.com/article/10.3390/v18080913/s1, Figure S1: Association between eugenol sulfate and large joint involvement; Figure S2: Association between eugenol sulfate and small joint involvement. Table S1: Baseline characteristics. Table S2: Metabolites; Table S3: KEGG pathway; Table S4: Fold-change; Table S5: Days from symptom onset to sample collection; Table S6: Correlation between 13 candidate metabolites and arthritis severity in CHIKF patients (*n* = 26).

## Abbreviations

The following abbreviations are used in this manuscript:

APTT	activated partial thromboplastin time
CHIKF	chikungunya fever
CHIKV	chikungunya virus
CK	creatine kinase
CRP	C-reactive protein
CV	coefficient of variation
DENV	dengue virus
DF	dengue fever
DHF	dengue hemorrhagic fever
eGFR	estimated glomerular filtration rate
FC	foldchange
GEE	generalized estimating equation
INR	international normalized ratio
KEGG	Kyoto Encyclopedia of Genes and Genomes
LC-MS/MS	liquid chromatography–tandem mass spectrometry
LysoPC	lysophosphatidylcholine
mTORC	mechanistic target of rapamycin complex
OPLS-DA	orthogonal partial least squares discriminant analysis
PCA	principal component analysis
PPAR	peroxisome proliferator-activated receptor
PT	prothrombin Time
QC	quality control
