# Supplementary material for: Metabolic Signatures of Chikungunya Versus Dengue: A Comparative Study
Source: Viruses. 2026 Aug 20;18(8):913. doi: 10.3390/v18080913 (PMC13517393; doi:10.3390/v18080913)
Supplement: Supplementary file 1 [file viruses-18-00913-s001.zip › Figure S2. Association between eugenol sulfate and small joint involvement.pdf]

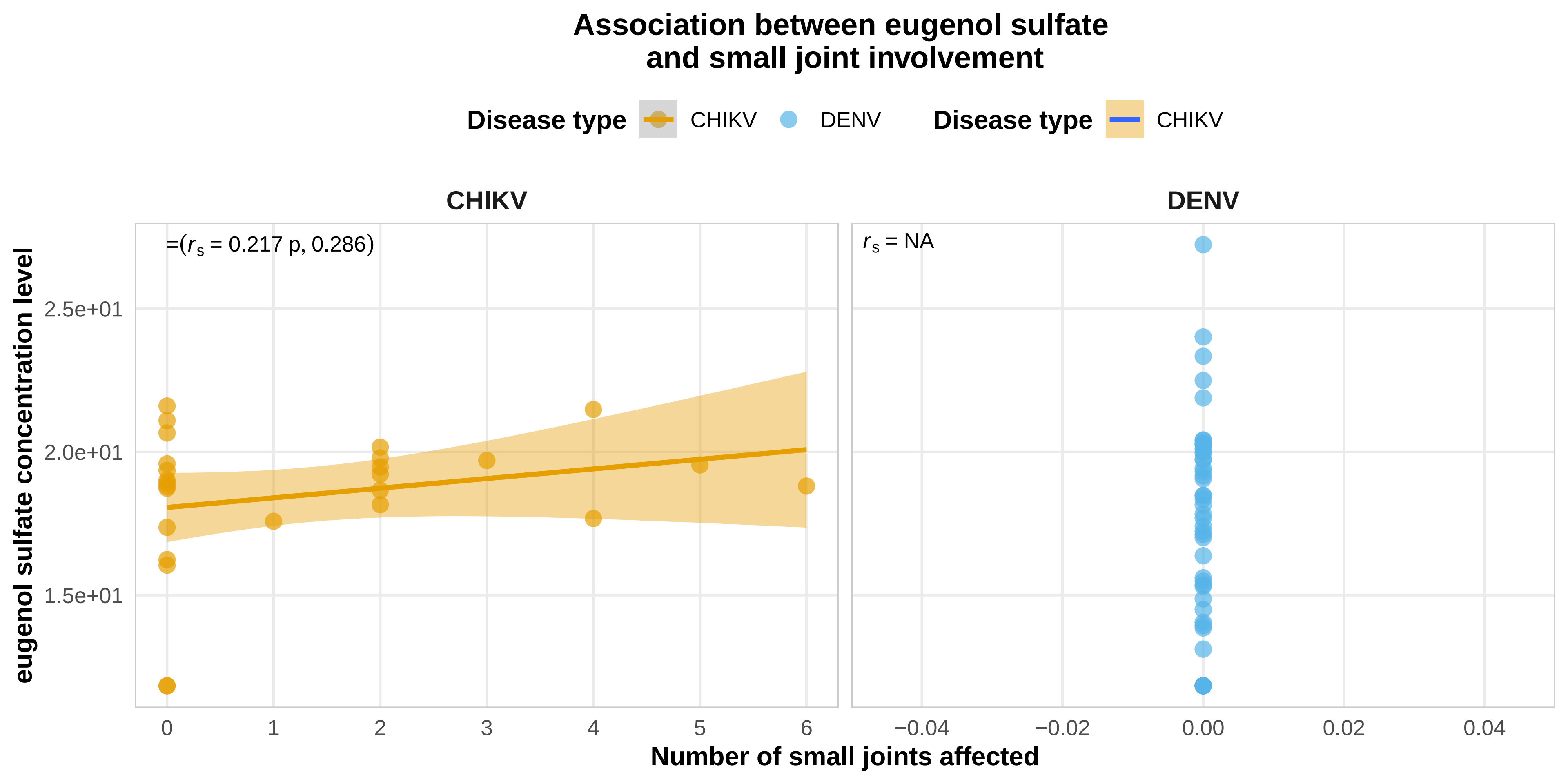
Association between eugenol sulfate
and small joint involvement
Disease type
CHIKV
DENV
Disease type
CHIKV
CHIKV
DENV
=(r_s = 0.217 p, 0.286)
r_s = NA
eugenol sulfate concentration level
2.5e+01
2.0e+01
1.5e+01
0
1
2
3
4
5
6
−0.04
−0.02
0.00
0.02
0.04
Number of small joints affected
