# Supplementary material for: Metabolic Signatures of Chikungunya Versus Dengue: A Comparative Study
Source: Viruses. 2026 Aug 20;18(8):913. doi: 10.3390/v18080913 (PMC13517393; doi:10.3390/v18080913)
Supplement: Supplementary file 1 [file viruses-18-00913-s001.zip › R package/1CDE.pdf]

1C

```
setwd('/Users/jiaojiao/Desktop/筛选后统计分析/figure1C')
library(ggplot2)
library(eoffice)
dt<-read.csv('raw_metabolites.csv',row.names = 1)

data<-as.data.frame(t(dt))
data$group<-c(rep("C",28),rep("D",47),rep("H",28))
count<- apply(data,2,function(c)sum(c!=0,na.rm = T))
a<-cbind(names(data),count)
write.csv(a,"sample_count.csv")
C<-data[which(data$group=="C"),-1245]
count=c()
data1<-C[1,]
sum<-colSums(data1,na.rm = T)
count<-sum(sum!=0,na.rm = T)
for (i in 2:nrow(C)) {
  data1<-C[c(1:i),]
  sum<-colSums(data1,na.rm = T)
  c<-sum(sum!=0,na.rm = T)
  count<-c(count,c)
}
count<-as.data.frame(count)
count$group<-rep('C')
count$number<-as.numeric(row.names(count))
count$number<-count$number /28


D<-data[which(data$group=="D"),-1245]
data1<-D[1,]
sum<-colSums(data1,na.rm = T)
count_A<-sum(sum!=0,na.rm = T)
for (i in 2:nrow(D)) {
  data1<-D[c(1:i),]
  sum<-colSums(data1,na.rm = T)
  c<-sum(sum!=0,na.rm = T)
  count_A<-c(count_A,c)
}
count_A<-as.data.frame(count_A)
count_A$group<-rep('D')
colnames(count_A)[1]<-'count'
count_A$number<-as.numeric(row.names(count_A))
count_A$number<-count_A$number / 47
```

```
H<-data[which(data$group=="H"),-1245]
count_P=c()
data1<-H[1,]
sum<-colSums(data1,na.rm = T)
count_P<-sum(sum!=0,na.rm = T)
for (i in 2:nrow(H)) {
  data1<-H[c(1:i),]
  sum<-colSums(data1,na.rm = T)
  c<-sum(sum!=0,na.rm = T)
  count_P<-c(count_P,c)
}
count_P<-as.data.frame(count_P)
count_P$group<-rep('H')
count_P$number<-as.numeric(row.names(count_P))
count_P$number<-count_P$number / 28
colnames(count_P)[1]<-'count'
count_dt<-rbind(count,count_P,count_A)
write.csv(count_dt,'Ord_count.csv')

data<-read.csv('Ord_count.csv')

p<-ggplot(data,aes(x=data$number,y=count,col=group,shape=group))+
  geom_point(aes(x=data$number,y=count,col=group,shape=group),size=4,alpha=0.9)+
  geom_line(size=1.5,alpha=0.7)+
  scale_shape_manual(values=c(17,16,18))+
  scale_color_manual(values = c("#3578AC","#EAC450","#D5231F"))+
  theme_classic()+
  theme(axis.title.x = element_blank(),
        axis.title.y = element_blank())

topptx(p, 'sample_metaboltes_count.pptx')
```

1D

```
setwd('/Users/jiaojiao/Desktop')
library(ggplot2)
library(eoffice)
library("readxl")
data <- read_excel("LOG10 后数据.xlsx")
cv <- apply(data[,3:16], 1, function(x) {
   mean_x <- mean(x, na.rm = TRUE)
  sd_x <- sd(x, na.rm = TRUE)
  if (mean_x != 0) {
    return(sd_x / mean_x)
  } else {
```

```
    return(NA)
  }
})
data$CV<-cv


p <- ggplot(data, aes(x = Metabolites, y = CV, fill = CV)) +
  geom_bar(stat = "identity", color = "transparent") +
  scale_fill_gradient(high = "yellow", low = "orange") +
  theme_minimal() +
  labs(
    x = "Metabolites",
    y = "CV VALUE"
  ) +
  theme_classic() +
  theme(
    axis.text.x = element_blank(),
    axis.ticks.x = element_blank(),
    plot.title = element_text(hjust = 0.5),
    plot.subtitle = element_text(hjust = 0.5)
  )
p
topptx(p,'QC_13cv.pptx')
1E
setwd('/Users/jiaojiao/Desktop')
library(ggplot2)
library(dplyr)
library("readxl")
metabolite_df <- read_excel("1E.xlsx")# 1. 加载必要的 R 包
superclass_count <- metabolite_df %>%
  count(`Super Class`, sort = TRUE) %>% # 注意：列名中有空格，需要用反引号``包裹
  rename(Count = n) # 将自动生成的计数列'n'重命名为更易懂的'Count'

# 查看统计结果
print(superclass_count)

# 4. 绘制水平条形图
ggplot(data = superclass_count,
       aes(x = Count,
           y = reorder(`Super Class`, Count))) +
  # reorder()函数是关键：它根据 Count 的值对 Super Class 进行排序
  geom_col(fill = "steelblue", width = 0.7) +
  # geom_col 用于绘制条形图，fill 设置填充颜色，width 设置条形宽度
  labs(title = "Classification and Distribution of Metabolites by Superclass",
```

```
        x = "Number of Metabolites",
        y = NULL) +
  # labs() 设置标题和坐标轴标签。将 y 轴标签设为 NULL 可以让图更简洁。
  theme_minimal() +
  # 使用一个简洁的图形主题
  theme(
    plot.title = element_text(hjust = 0.5, size = 14, face = "bold"), # 标题居中、加大、加粗
    axis.text.y = element_text(size = 10),     # 设置 y 轴（类别名）文字大小
    axis.title.x = element_text(size = 12),   # 设置 x 轴标题文字大小
    panel.grid.major.y = element_blank(),      # 移除水平主网格线，使图表更清晰
    panel.grid.minor.x = element_blank()       # 移除垂直次要网格线
  )

# 5. （可选）将图片保存到本地
# ggsave("My_Metabolite_SuperClass_Distribution.png",
#             width = 10,   # 图片宽度（英寸）
#             height = 6,   # 图片高度（英寸）
#             dpi = 300)    # 分辨率
```
