# Supplementary material for: Metabolic Signatures of Chikungunya Versus Dengue: A Comparative Study
Source: Viruses. 2026 Aug 20;18(8):913. doi: 10.3390/v18080913 (PMC13517393; doi:10.3390/v18080913)
Supplement: Supplementary file 1 [file viruses-18-00913-s001.zip › R package/2BCDE.pdf]

```
rm(list=ls())

setwd("/Users/jiaojiao/Desktop/筛选后统计分析/figure3BCDE")
data <- read.csv("364sample-185metabolites-log10-auto.csv", row.names = 1)
clin <- read.csv('Cinformation.csv')
origin <- read.csv("364sample_185metabolites.csv",check.names = F,row.names = 1)
F <- clin$sample[clin$GENDER == 'F']
M <- clin$sample[clin$GENDER == 'M']

F <- intersect(F, colnames(data))
M <- intersect(M, colnames(data))


result_gender <- data.frame()

for (Metabolites in row.names(data)) {
  group_F <- as.numeric(data[Metabolites, F])
  group_M <- as.numeric(data[Metabolites, M])
  result <- wilcox.test(group_F, group_M)
  p <- result$p.value
  log10.fc <- mean(group_F)-mean(group_M)

  group_F <- as.numeric(origin[Metabolites, F])
  group_M <- as.numeric(origin[Metabolites, M])

  NA_F <- sum(is.na(group_F))
  NA_M <- sum(is.na(group_M))
  nonNA_F <- length(group_F) - NA_F
  nonNA_M <- length(group_M) - NA_M

  contingency_table <- matrix(c(nonNA_F, NA_F, nonNA_M, NA_M), nrow = 2,
                              dimnames = list(Status = c("NonNA", "NA"), Gender =
c("F", "M")))


  if (any(contingency_table < 5)) {
    test_result <- fisher.test(contingency_table)
    test_used <- "Fisher"
  } else {
    test_result <- chisq.test(contingency_table)
    test_used <- "Chi-square"
  }

  result_row <- data.frame(
```

```
    metabolic = Metabolites,
    p.value = p,
    log10.fc = log10.fc,
    N_F_nonNA = nonNA_F,
    N_M_nonNA = nonNA_M,
    detected_F = nonNA_F/length(group_F),
    detected_M = nonNA_M/length(group_M),
    NA_test_pvalue = test_result$p.value,
    NA_test_used = test_used
  )

  result_gender <- rbind(result_gender, result_row)
}

write.csv(result_gender, 'gender-wilcox-new.csv', row.names = FALSE)

####plot C####
data1<-read.csv('gender-wilcox-new.csv')
data1$color<- "no"
data1$color[(data1$p.value<0.05)&(data1$log10.fc>log10(1.2))] <- "up"
data1$color[(data1$p.value<0.05)&(data1$log10.fc<(-log10(1.2)))] <-"down"
table(data1$color)
library(ggplot2)
library(ggrepel)
top_10_indices <- order(data1$p.value)[1:10]
significant_points <- data.frame(
  log10.fc = data1$log10.fc[top_10_indices],
  p.value = -log10(data1$p.value[top_10_indices]),
  metabolic = data1$metabolic[top_10_indices]
)
gg <- ggplot(data1, aes(x = -log10.fc, y = -log10(data1$p.value), color = data1$color)) +
  geom_point(size = 3) +
  scale_color_manual(values = c('down' = '#DE3024', 'up' = '#009ACD', 'no' = 'grey')) +
  geom_vline(xintercept = log10(1.2), linetype = 2, color = "grey", size = 1) +
  geom_vline(xintercept = -log10(1.2), linetype = 2, color = "grey", size = 1) +
  geom_hline(yintercept = -log10(0.05), linetype = 2, color = "grey", size = 1) +

  theme(
    panel.background = element_rect(fill = 'transparent', color = 'transparent'),
    panel.grid.major = element_blank(), #
    panel.grid.minor = element_blank(), #
    axis.line.x = element_line(size = 0.5), #
    axis.line.y = element_line(size = 0.5), #
    axis.text = element_text(size = 6), #
```

```
    axis.ticks = element_line(size = 1),

    legend.position = "none"
  ) +
  labs(
    x = "Log10(male/female)",
    y = "-Log10(p value)",
    color = "",
    size=8
  )+ geom_text_repel(
    data = significant_points,
    aes(x = -log10.fc, y = p.value, label = metabolic),
    size = 5,
    color = "black",
    box.padding = unit(0.4, "lines"),
    segment.color = "black",
    segment.size = 0.6,
    min.segment.length = 0.5
  )+
  theme_minimal(base_size = 16) +
  theme(
    panel.grid.major = element_blank(),
    panel.grid.minor = element_blank(),
    axis.line = element_line(color = "black"),
    axis.title = element_text(color = "black", size = 16),
    axis.ticks   = element_line(color = "black", linewidth = 0.5),
    axis.text = element_text(color = "black", size = 15),
    legend.position = 'none'
  )
gg
ggsave("C_gender_wilcox.pdf", plot = gg, height = 4.5, width = 4.5)
#D 组
rm(list=ls())

setwd("/Users/jiaojiao/Desktop/筛选后统计分析/figure3BCDE")
data <- read.csv("364sample-185metabolites-log10-auto.csv", row.names = 1)

clin <- read.csv('Dinformation.csv')
origin <- read.csv("364sample_185metabolites.csv",check.names = F,row.names = 1)
F <- clin$sample[clin$GENDER == 'F']
M <- clin$sample[clin$GENDER == 'M']

F <- intersect(F, colnames(data))
M <- intersect(M, colnames(data))
```

```
result_gender <- data.frame()

for (Metabolites in row.names(data)) {
  group_F <- as.numeric(data[Metabolites, F])
  group_M <- as.numeric(data[Metabolites, M])
  result <- wilcox.test(group_F, group_M)
  p <- result$p.value
  log10.fc <- mean(group_F)-mean(group_M)

  group_F <- as.numeric(origin[Metabolites, F])
  group_M <- as.numeric(origin[Metabolites, M])

  NA_F <- sum(is.na(group_F))
  NA_M <- sum(is.na(group_M))
  nonNA_F <- length(group_F) - NA_F
  nonNA_M <- length(group_M) - NA_M

  contingency_table <- matrix(c(nonNA_F, NA_F, nonNA_M, NA_M), nrow = 2,
                              dimnames = list(Status = c("NonNA", "NA"), Gender =
c("F", "M")))


  if (any(contingency_table < 5)) {
    test_result <- fisher.test(contingency_table)
    test_used <- "Fisher"
  } else {
    test_result <- chisq.test(contingency_table)
    test_used <- "Chi-square"
  }

  result_row <- data.frame(
    metabolic = Metabolites,
    p.value = p,
    log10.fc = log10.fc,
    N_F_nonNA = nonNA_F,
    N_M_nonNA = nonNA_M,
    detected_F = nonNA_F/length(group_F),
    detected_M = nonNA_M/length(group_M),
    NA_test_pvalue = test_result$p.value,
    NA_test_used = test_used
  )
```

```
  result_gender <- rbind(result_gender, result_row)
}

write.csv(result_gender, 'D-gender-wilcox.csv', row.names = FALSE)

####plotD####
library(ggplot2)
library(ggrepel)
data1<-read.csv('D-gender-wilcox.csv')
data1$color<- "no"
data1$color[(data1$p.value<0.05)&(data1$log10.fc>log10(1.2))] <- "up"
data1$color[(data1$p.value<0.05)&(data1$log10.fc<(-log10(1.2)))] <-"down"
table(data1$color)
top_10_indices <- order(data1$p.value)[1:10]
significant_points <- data.frame(
  log10.fc = data1$log10.fc[top_10_indices],
  p.value = -log10(data1$p.value[top_10_indices]),
  metabolic = data1$metabolic[top_10_indices]
)
gg <- ggplot(data1, aes(x = -log10.fc, y = -log10(data1$p.value), color = data1$color)) +
  geom_point(size = 3) +
  scale_color_manual(values = c('down' = '#DE3024', 'up' = '#009ACD', 'no' = 'grey')) +
  geom_vline(xintercept = log10(1.2), linetype = 2, color = "grey", size = 1) +
  geom_vline(xintercept = -log10(1.2), linetype = 2, color = "grey", size = 1) +
  geom_hline(yintercept = -log10(0.05), linetype = 2, color = "grey", size = 1) +

  theme(
    panel.background = element_rect(fill = 'transparent', color = 'transparent'),
    panel.grid.major = element_blank(), #
    panel.grid.minor = element_blank(), #
    axis.line.x = element_line(size = 0.5), #
    axis.line.y = element_line(size = 0.5), #
    axis.text = element_text(size = 6), #
    axis.ticks = element_line(size = 1),

    legend.position = "none"
  ) +
  labs(
    x = "Log10(male/female)",
    y = "-Log10(p value)",
    color = "",
    size=8
  )+ geom_text_repel(
    data = significant_points,
```

```
    aes(x = -log10.fc, y = p.value, label = metabolic),
    size = 5,
    color = "black",
    box.padding = unit(0.4, "lines"),
    segment.color = "black",
    segment.size = 0.6,
    min.segment.length = 0.5
  )+
  theme_minimal(base_size = 16) +
  theme(
    panel.grid.major = element_blank(),
    panel.grid.minor = element_blank(),
    axis.line = element_line(color = "black"),
    axis.title = element_text(color = "black", size = 16),
    axis.ticks   = element_line(color = "black", linewidth = 0.5),
    axis.text = element_text(color = "black", size = 15),
    legend.position = 'none'
  )
gg
ggsave("D_gender_wilcox.pdf", plot = gg, height = 4.5, width = 4.5)

#H 组
rm(list=ls())

setwd("/Users/jiaojiao/Desktop/筛选后统计分析/figure3BCDE")
data <- read.csv("364sample-185metabolites-log10-auto.csv", row.names = 1)

clin <- read.csv('Hinformation.csv')
origin <- read.csv("364sample_185metabolites.csv",check.names = F,row.names = 1)
F <- clin$sample[clin$GENDER == 'F']
M <- clin$sample[clin$GENDER == 'M']

F <- intersect(F, colnames(data))
M <- intersect(M, colnames(data))


result_gender <- data.frame()

for (Metabolites in row.names(data)) {
  group_F <- as.numeric(data[Metabolites, F])
  group_M <- as.numeric(data[Metabolites, M])
  result <- wilcox.test(group_F, group_M)
  p <- result$p.value
  log10.fc <- mean(group_F)-mean(group_M)
```

```
    group_F <- as.numeric(origin[Metabolites, F])
    group_M <- as.numeric(origin[Metabolites, M])

    NA_F <- sum(is.na(group_F))
    NA_M <- sum(is.na(group_M))
    nonNA_F <- length(group_F) - NA_F
    nonNA_M <- length(group_M) - NA_M

    contingency_table <- matrix(c(nonNA_F, NA_F, nonNA_M, NA_M), nrow = 2,
                                dimnames = list(Status = c("NonNA", "NA"), Gender =
c("F", "M")))


    if (any(contingency_table < 5)) {
      test_result <- fisher.test(contingency_table)
      test_used <- "Fisher"
    } else {
      test_result <- chisq.test(contingency_table)
      test_used <- "Chi-square"
    }

    result_row <- data.frame(
      metabolic = Metabolites,
      p.value = p,
      log10.fc = log10.fc,
      N_F_nonNA = nonNA_F,
      N_M_nonNA = nonNA_M,
      detected_F = nonNA_F/length(group_F),
      detected_M = nonNA_M/length(group_M),
      NA_test_pvalue = test_result$p.value,
      NA_test_used = test_used
    )

    result_gender <- rbind(result_gender, result_row)
}

write.csv(result_gender, 'H-gender-wilcox.csv', row.names = FALSE)

####plotH####
library(ggplot2)
library(ggrepel)
data1<-read.csv('H-gender-wilcox.csv')
data1$color<- "no"
```

```
data1$color[(data1$p.value<0.05)&(data1$log10.fc>log10(1.2))] <- "up"
data1$color[(data1$p.value<0.05)&(data1$log10.fc<(-log10(1.2)))] <-"down"
table(data1$color)
top_10_indices <- order(data1$p.value)[1:10]
significant_points <- data.frame(
  log10.fc = data1$log10.fc[top_10_indices],
  p.value = -log10(data1$p.value[top_10_indices]),
  metabolic = data1$metabolic[top_10_indices]
)
gg <- ggplot(data1, aes(x = -log10.fc, y = -log10(data1$p.value), color = data1$color)) +
  geom_point(size = 3) +
  scale_color_manual(values = c('down' = '#DE3024', 'up' = '#009ACD', 'no' = 'grey')) +
  geom_vline(xintercept = log10(1.2), linetype = 2, color = "grey", size = 1) +
  geom_vline(xintercept = -log10(1.2), linetype = 2, color = "grey", size = 1) +
  geom_hline(yintercept = -log10(0.05), linetype = 2, color = "grey", size = 1) +

  theme(
    panel.background = element_rect(fill = 'transparent', color = 'transparent'),
    panel.grid.major = element_blank(), #
    panel.grid.minor = element_blank(), #
    axis.line.x = element_line(size = 0.5), #
    axis.line.y = element_line(size = 0.5), #
    axis.text = element_text(size = 6), #
    axis.ticks = element_line(size = 1),

    legend.position = "none"
  ) +
  labs(
    x = "Log10(male/female)",
    y = "-Log10(p value)",
    color = "",
    size=8
  )+ geom_text_repel(
    data = significant_points,
    aes(x = -log10.fc, y = p.value, label = metabolic),
    size = 5,
    color = "black",
    box.padding = unit(0.4, "lines"),
    segment.color = "black",
    segment.size = 0.6,
    min.segment.length = 0.5
  )+
  theme_minimal(base_size = 16) +
  theme(
```

```
      panel.grid.major = element_blank(),
      panel.grid.minor = element_blank(),
      axis.line = element_line(color = "black"),
      axis.title = element_text(color = "black", size = 16),
      axis.ticks    = element_line(color = "black", linewidth = 0.5),
      axis.text = element_text(color = "black", size = 15),
      legend.position = 'none'
  )
gg
ggsave("H_gender_wilcox.pdf", plot = gg, height = 4.5, width = 4.5)


#age 部分
library('ggvenn')
C<-read.csv('gender-wilcox-new.csv')
C$color<- "no"
C$color[(C$p.value<0.05)&(C$log10.fc>log10(1.2))] <- "up"
C$color[(C$p.value<0.05)&(C$log10.fc<(-log10(1.2)))] <-"down"
table(C$color)
C_negative <- C$metabolic[C$color == 'down']

D<-read.csv('D-gender-wilcox.csv')
D$color<- "no"
D$color[(D$p.value<0.05)&(D$log10.fc>log10(1.2))] <- "up"
D$color[(D$p.value<0.05)&(D$log10.fc<(-log10(1.2)))] <-"down"
table(D$color)
D_negative <- D$metabolic[D$color == 'down']

H<-read.csv('H-gender-wilcox.csv')
H$color<- "no"
H$color[(H$p.value<0.05)&(H$log10.fc>log10(1.2))] <- "up"
H$color[(H$p.value<0.05)&(H$log10.fc<(-log10(1.2)))] <-"down"
table(H$color)
H_negative <- H$metabolic[H$color == 'down']
venn_list <- list(
  "C" = C_negative,
  "D" = D_negative,
  'H'=H_negative
)
library(purrr)
intersect(C_negative,D_negative)
common_metabolic<- reduce(venn_list, intersect)
library(eoffice)
p1 <- ggvenn(venn_list,
```

```
                fill_color = c("#DE3024", "#009ACD", "#FFD700"),
                stroke_size = 0.5, set_name_size = 4)

topptx(p1,filename = "intersect_lm_test.pptx")
####intersect CD and gender####
library(readr)

CD_GEEglm_meta <- read_csv("CD_GEEglm_meta.csv")
CD_pos<-CD_GEEglm_meta$Metabolite[CD_GEEglm_meta$Estimate>0&CD_GEEglm_
meta$p_value<0.05]
common<-intersect(C_negative,CD_pos)

write.csv(common,'increased in male and C.csv')
```
