# Supplementary material for: Metabolic Signatures of Chikungunya Versus Dengue: A Comparative Study
Source: Viruses. 2026 Aug 20;18(8):913. doi: 10.3390/v18080913 (PMC13517393; doi:10.3390/v18080913)
Supplement: Supplementary file 1 [file viruses-18-00913-s001.zip › R package/3CDE.pdf]

2CDE

```
setwd('/Users/jiaojiao/Desktop/筛选后统计分析/figure2CDEF')
library(geepack)
library(readxl)
rm(list = ls())
Cinformation <- read_excel("Cinformation.xlsx")
Dinformation <- read_excel("Dinformation.xlsx")
Hinformation <- read_excel("Hinformation.xlsx")
Cinformation$Group <- "C"
Dinformation$Group <- "D"
Hinformation$group <- "H"
metabolites_data <- read.csv("log10 预处理.csv",row.names = 1)
metabolites_data<-as.data.frame(t(metabolites_data))
metabolites_data$Sample<-row.names(metabolites_data)

# 只保留需要的列（确保列名一致）
C_sub <- Cinformation[, c("Sample", 'Gender','Age','病程', "BP", "糖尿病", "冠心病", "Group")]
colnames(C_sub)[2:3]<-c('Gender','Age')
D_sub <- Dinformation[, c("Sample", 'Gender','Age','病程', "BP", "糖尿病", "冠心病", "Group")]
H_sub <- Hinformation[, c("Sample", 'Gender','Age','病程', "BP", "糖尿病", "冠心病", "Group")]

combined_data <- rbind(C_sub, D_sub, H_sub)
####C vs D####
CD_df<-combined_data[combined_data$Group%in%c('C','D'),]
CD_df<-merge(CD_df,metabolites_data,by='Sample',all.x = TRUE)
CD_df$Group <- as.numeric(ifelse(CD_df$Group == "C", 1,
                                   ifelse(CD_df$Group == "D", 0, CD_df$Group)))
CD_df$gender <- as.numeric( ifelse(CD_df$Gender == "f", 1,
                                     ifelse(CD_df$Gender == "m", 0, CD_df$Gender)))
table(CD_df$Group)
table(CD_df$Gender)
CD_df$ID<-c(1:nrow(CD_df))
metabolite_list<-colnames(CD_df)[9:1253]
CD_results_list <- list()

failed_metabolites <- c()

for (meta in metabolite_list) {
    cat("正在分析代谢物:", meta, "\n")
```

```
    tryCatch({
        # 创建完整的数据子集（删除当前代谢物和相关变量的缺失值）
        required_vars <- c("ID", "Group", "Gender","Age", "病程", "BP", "糖尿病", "冠心病
", meta)
        temp_df <- CD_df[complete.cases(CD_df[required_vars]), ]
        temp_df$病程_numeric <- as.numeric(temp_df$病程)
        temp_df$Age_numeric <- as.numeric(temp_df$Age)


        # 检查数据是否有效
        if (nrow(temp_df) == 0) {
            stop("无有效观测值")
        }

        # 检查 Group 变量的变异性
        if (length(unique(temp_df$Group)) < 2) {
            stop("Group 变量缺乏变异性")
        }

        # 构建公式
        formula <- as.formula(paste0("`", meta, "` ~ Group + Gender + Age+病程 + BP +
糖尿病 + 冠心病"))

        # 拟合 GEE 模型
        model <- geeglm(formula,
                        data = temp_df,
                        id = ID)

        # 提取结果
        coef_summary <- summary(model)$coefficients

        # 确保有足够的行
        if (nrow(coef_summary) >= 2) {
            est <- coef_summary[2, "Estimate"]
            se   <- coef_summary[2, "Std.err"]
            ci_lower <- if (!is.na(se)) est - 1.96 * se else NA
            ci_upper <- if (!is.na(se)) est + 1.96 * se else NA
            p_value <- coef_summary[2, "Pr(>|W|)"]

            CD_results_list[[meta]] <- data.frame(
                Metabolite = meta,
                Estimate = est,
                Std.err = se,
```

```
                    CI_lower = ci_lower,
                    CI_upper = ci_upper,
                    p_value = p_value,
                    n_obs = nrow(temp_df),
                    stringsAsFactors = FALSE
                )

                cat("   ✓ 完成，样本数:", nrow(temp_df), "\n")
            } else {
                stop("模型系数不足")
            }

        }, error = function(e) {
            cat("   ✗ 错误:", e$message, "\n")
            failed_metabolites <<- c(failed_metabolites, meta)
            CD_results_list[[meta]] <- data.frame(
                Metabolite = meta,
                Estimate = NA,
                Std.err = NA,
                CI_lower = NA,
                CI_upper = NA,
                p_value = NA,
                n_obs = 0,
                error_msg = e$message,
                stringsAsFactors = FALSE
            )
        })
}


CD_final_results <- do.call(rbind, CD_results_list)
CD_final_results$sig <-ifelse(CD_final_results$p_value<0.05, 'sig','not sig')
table(CD_final_results$sig)

write.csv(CD_final_results,'CD_GEEglm_meta.csv')

####C vs H####
CH_df<-combined_data[combined_data$Group%in%c('C','H'),]
CH_df<-merge(CH_df,metabolites_data,by='Sample',all.x = TRUE)
CH_df$Group <- as.numeric(ifelse(CH_df$Group == "C", 1,
                                 ifelse(CH_df$Group == "H", 0, CH_df$Group)))
CH_df$gender <- as.numeric( ifelse(CH_df$Gender == "f", 1,
                                   ifelse(CH_df$Gender == "m", 0, CH_df$Gender)))
table(CH_df$Group)
```

```
table(CH_df$Gender)
CH_df$ID<-c(1:nrow(CH_df))
metabolite_list<-colnames(CH_df)[9:1253]
CH_results_list <- list()

failed_metabolites <- c()

for (meta in metabolite_list) {
	cat("正在分析代谢物:", meta, "\n")

	tryCatch({
		# 创建完整的数据子集（删除当前代谢物和相关变量的缺失值）
		required_vars <- c("ID", "Group", "Gender", "Age", meta)
		temp_df <- CH_df[complete.cases(CH_df[required_vars]), ]
		temp_df$Age_numeric <- as.numeric(temp_df$Age)


		# 检查数据是否有效
		if (nrow(temp_df) == 0) {
			stop("无有效观测值")
		}

		# 检查 Group 变量的变异性
		if (length(unique(temp_df$Group)) < 2) {
			stop("Group 变量缺乏变异性")
		}

		# 构建公式
		formula <- as.formula(paste0("`", meta, "` ~ Group + Gender+Age "))

		# 拟合 GEE 模型
		model <- geeglm(formula,
						data = temp_df,
						id = ID)

		# 提取结果
		coef_summary <- summary(model)$coefficients

		# 确保有足够的行
		if (nrow(coef_summary) >= 2) {
			est <- coef_summary[2, "Estimate"]
			se   <- coef_summary[2, "Std.err"]
			ci_lower <- if (!is.na(se)) est - 1.96 * se else NA
			ci_upper <- if (!is.na(se)) est + 1.96 * se else NA
```

```
            p_value <- coef_summary[2, "Pr(>|W|)"]

            CH_results_list[[meta]] <- data.frame(
                Metabolite = meta,
                Estimate = est,
                Std.err = se,
                CI_lower = ci_lower,
                CI_upper = ci_upper,
                p_value = p_value,
                n_obs = nrow(temp_df),
                stringsAsFactors = FALSE
            )

            cat("    ✓ 完成，样本数:", nrow(temp_df), "\n")
        } else {
            stop("模型系数不足")
        }

    }, error = function(e) {
        cat("    ✗ 错误:", e$message, "\n")
        failed_metabolites <<- c(failed_metabolites, meta)
        CH_results_list[[meta]] <- data.frame(
            Metabolite = meta,
            Estimate = NA,
            Std.err = NA,
            CI_lower = NA,
            CI_upper = NA,
            p_value = NA,
            n_obs = 0,
            error_msg = e$message,
            stringsAsFactors = FALSE
        )
    })
}


CH_final_results <- do.call(rbind, CH_results_list)
CH_final_results$sig <-ifelse(CH_final_results$p_value<0.05, 'sig','not sig')
table(CH_final_results$sig)

write.csv(CH_final_results,'CH_GEEglm_meta.csv')
####D vs H####
DH_df<-combined_data[combined_data$Group%in%c('D','H'),]
DH_df<-merge(DH_df,metabolites_data,by='Sample',all.x = TRUE)
```

```
DH_df$Group <- as.numeric(ifelse(DH_df$Group == "D", 1,
                                  ifelse(DH_df$Group == "H", 0, DH_df$Group)))
DH_df$gender <- as.numeric( ifelse(DH_df$Gender == "f", 1,
                                    ifelse(DH_df$Gender == "m", 0, DH_df$Gender)))
table(DH_df$Group)
table(DH_df$Gender)
DH_df$ID<-c(1:nrow(DH_df))
metabolite_list<-colnames(DH_df)[9:1253]
DH_results_list <- list()

failed_metabolites <- c()

for (meta in metabolite_list) {
    cat("正在分析代谢物:", meta, "\n")

    tryCatch({
        # 创建完整的数据子集（删除当前代谢物和相关变量的缺失值）
        required_vars <- c("ID", "Group", "Gender", "Age", meta)
        temp_df <- DH_df[complete.cases(DH_df[required_vars]), ]
        temp_df$Age_numeric <- as.numeric(temp_df$Age)

        # 检查数据是否有效
        if (nrow(temp_df) == 0) {
            stop("无有效观测值")
        }

        # 检查 Group 变量的变异性
        if (length(unique(temp_df$Group)) < 2) {
            stop("Group 变量缺乏变异性")
        }

        # 构建公式
        formula <- as.formula(paste0("`", meta, "` ~ Group + Gender +Age"))

        # 拟合 GEE 模型
        model <- geeglm(formula,
                        data = temp_df,
                        id = ID)

        # 提取结果
        coef_summary <- summary(model)$coefficients

        # 确保有足够的行
        if (nrow(coef_summary) >= 2) {
```

```
            est <- coef_summary[2, "Estimate"]
            se    <- coef_summary[2, "Std.err"]
            ci_lower <- if (!is.na(se)) est - 1.96 * se else NA
            ci_upper <- if (!is.na(se)) est + 1.96 * se else NA
            p_value <- coef_summary[2, "Pr(>|W|)"]

            DH_results_list[[meta]] <- data.frame(
                Metabolite = meta,
                Estimate = est,
                Std.err = se,
                CI_lower = ci_lower,
                CI_upper = ci_upper,
                p_value = p_value,
                n_obs = nrow(temp_df),
                stringsAsFactors = FALSE
            )

            cat("    ✓ 完成，样本数:", nrow(temp_df), "\n")
        } else {
            stop("模型系数不足")
        }

    }, error = function(e) {
        cat("    ✗ 错误:", e$message, "\n")
        failed_metabolites <<- c(failed_metabolites, meta)
        DH_results_list[[meta]] <- data.frame(
            Metabolite = meta,
            Estimate = NA,
            Std.err = NA,
            CI_lower = NA,
            CI_upper = NA,
            p_value = NA,
            n_obs = 0,
            error_msg = e$message,
            stringsAsFactors = FALSE
        )
    })
}


DH_final_results <- do.call(rbind, DH_results_list)
DH_final_results$sig <-ifelse(DH_final_results$p_value<0.05, 'sig','not sig')
table(DH_final_results$sig)
```

```
write.csv(DH_final_results,'DH_GEEglm_meta.csv')
#绘图
library(ggplot2)
library(ggrepel)
library(scales)
squash_axis <- function(from, to, factor) {

  trans <- function(x) {
    # Initialize a logical vector to identify NAs
    is_na <- is.na(x)

    # get indices for the relevant regions
    isq <- !is_na & x > from & x < to
    ito <- !is_na & x >= to

    # apply transformation
    x[isq] <- from + (x[isq] - from)/factor
    x[ito] <- from + (to - from)/factor + (x[ito] - to)

    return(x)
  }

  inv <- function(x) {
    # Initialize a logical vector to identify NAs
    is_na <- is.na(x)

    # get indices for the relevant regions
    isq <- !is_na & x > from & x < from + (to - from)/factor
    ito <- !is_na & x >= from + (to - from)/factor

    # apply transformation
    x[isq] <- from + (x[isq] - from) * factor
    x[ito] <- to + (x[ito] - (from + (to - from)/factor))

    return(x)
  }

  # return the transformation and inverse transformation
  return(scales::trans_new("squash_axis", trans, inv))
}
####plot CD####
data1<-read.csv('CD_GEEglm_meta.csv',row.names = 1)
data1$logp          <- -log10(data1$p_value)
finite_vals        <- data1$logp[is.finite(data1$logp)]
```

```
max_finite          <- if (length(finite_vals)>0) max(finite_vals, na.rm=TRUE) else 0
cap                 <- max_finite + 1
data1$logp_capped <- ifelse(is.infinite(data1$logp), cap, data1$logp)
data1$color<- "no"
data1$color[(data1$p_value<0.05)&(data1$Estimate>0)] <- "positive"
data1$color[(data1$p_value<0.05)&(data1$Estimate<0)]    <- "negative"
table(data1$color)

extreme_points <- subset(data1, is.infinite(logp))
normal_points    <- subset(data1, is.finite(logp))

extreme_points <- extreme_points[order(abs(extreme_points$Estimate)), ]

n_ext <- nrow(extreme_points)
if(n_ext > 0) {

  y_min_ext <- cap*1.01
  y_max_ext <- cap *1.28

  extreme_points$y_special <- seq(from = y_min_ext, to = y_max_ext, length.out =
n_ext)
} else {
  extreme_points$y_special <- numeric(0)
}


top_10_indices <- order(-abs(data1$Estimate))[1:10]
top_10_meta       <- data1$Metabolite[top_10_indices]

significant_points <- data.frame(
  estimate = data1$Estimate[top_10_indices],
  logp        = sapply(top_10_meta, function(m) {

    orig_logp <- data1$logp[ data1$Metabolite == m ]
    if (is.infinite(orig_logp)) {

      return(extreme_points$y_special[ match(m, extreme_points$Metabolite) ])
    } else {

      return(orig_logp)
    }
  }),
  metabolic = top_10_meta,
  stringsAsFactors = FALSE
```

```
)

y_tick_max <- floor(cap)

# 定义 squash_axis 函数
squash_axis <- function(from, to, factor) {
    # 这是一个坐标轴压缩函数，常用于处理异常值
    trans <- function(x) {
        # 如果值在[from, to]范围内，保持原值
        # 如果值超出范围，进行压缩处理
        ifelse(x >= from & x <= to, x,
               ifelse(x < from, from + (x - from) / factor,
                      to + (x - to) / factor))
    }

    inv <- function(x) {
        ifelse(x >= from & x <= to, x,
               ifelse(x < from, from + (x - from) * factor,
                      to + (x - to) * factor))
    }

    # 返回变换函数
    scales::trans_new("squash_axis", trans, inv)
}


gg <- ggplot() +


  geom_rect(aes(xmin = -Inf, xmax = Inf,
                ymin = cap,
                ymax = cap * 1.3),
            fill = "grey80", alpha = 0.5) +
  geom_hline(yintercept = -log10(0.05), linetype = 2, color = "black", size = 1) +

  # 5.2 绘制“普通”点：y 用 logp_capped，x 用 Estimate
  geom_point(data = normal_points,
             aes(x = Estimate, y = logp_capped, color = color),
             size = 3) +

  # 5.3 绘制“被截断”的点：用 y_special 作为纵坐标
  geom_point(data = extreme_points,
             aes(x = Estimate, y = y_special, color = color),
             size = 3) +
```

```
# 5.4 高亮前 10 个显著点（如果属于截断，用 y_special；否则用 logp_capped）
geom_point(data = significant_points,
           aes(x = estimate, y = logp),
           color = "gold", size = 4) +

# 5.5 在灰色区块下方画一条虚线，提示“所有 p 极小，集中展示”


# 5.6 给前 10 个显著点加标签
geom_text_repel(data = significant_points,
                aes(x = estimate, y = logp, label = metabolic),
                size = 5,                    # 调大标签字体
                color = "black",
                box.padding    = unit(0.4, "lines"),
                segment.color = "black",
                segment.size   = 0.6,
                min.segment.length = 0.5) +

# 5.7 美化：自定义配色、去网格、统一字体黑色并增大
scale_color_manual(values = c(
  'positive' = '#DE3024',
  'negative' = '#25108f',
  'no'         = 'grey'
)) +

# ** 关键：y 轴上限直接设为 cap * 1.3，与灰色区块 top 对齐 **
scale_y_continuous(
  limits = c(0, cap * 1.3),   # 下限可以从 0 或最小 logp 开始
  expand = c(0, 0) ,
  breaks = seq(0, y_tick_max, by = 4)# 去掉额外留白
) +

# 5.8 主题设置：所有文字黑色，字体统一变大
theme(
  plot.margin = margin(),
  panel.background    = element_rect(fill = 'transparent', color = 'transparent'),
  panel.grid.major    = element_blank(),
  panel.grid.minor    = element_blank(),
  axis.line.x          = element_line(size = 0.7, color = "black"),
  axis.line.y          = element_line(size = 0.7, color = "black"),
  axis.text             = element_text(size = 14, color = "black"),    # 刻度文字
  axis.title            = element_text(size = 16, color = "black"),    # 坐标轴标题
  axis.ticks            = element_line(size = 0.7, color = "black"),
```

```r
    legend.position       = "none",
    plot.title            = element_text(size = 18, color = "black", face = "bold")
  ) +

  # 5.9 坐标轴及标题
  labs(
    x = "Coefficient",
    y = "-log10(p value)",
    title = ""
  )+
  coord_trans(y = squash_axis(0, cap, 3))

gg
ggsave("CD_geeglm_meta.pdf", width = 5, height = 6)

####plot CH####
data1<-read.csv('CH_GEEglm_meta.csv',row.names = 1)
data1$logp           <- -log10(data1$p_value)
finite_vals        <- data1$logp[is.finite(data1$logp)]
max_finite         <- if (length(finite_vals)>0) max(finite_vals, na.rm=TRUE) else 0
cap                <- max_finite + 1   # 设定灰色区块的上界
data1$logp_capped <- ifelse(is.infinite(data1$logp), cap, data1$logp)
data1$color<- "no"
data1$color[(data1$p_value<0.05)&(data1$Estimate>0)] <- "positive"
data1$color[(data1$p_value<0.05)&(data1$Estimate<0)]   <- "negative"
table(data1$color)
# —— 2. 找到所有被截断的点（p 极小）和普通点 ——
extreme_points <- subset(data1, is.infinite(logp))      # 原始 logp 为 Inf 的那些行
normal_points   <- subset(data1, is.finite(logp))        # logp 有限的行

# —— 3. 给被截断点设计一个“美观的 y 坐标” ——
#       根据 Estimate 的绝对值排序，然后在区块内均匀分配 y 值
extreme_points <- extreme_points[order(abs(extreme_points$Estimate)), ]

n_ext <- nrow(extreme_points)
if(n_ext > 0) {
  # 目标区间 [cap * 0.8, cap * 0.98]，你可以根据需要调节
  y_min_ext <- cap*1.01
  y_max_ext <- cap *1.28

  # 在这个范围内等距分配
  extreme_points$y_special <- seq(from = y_min_ext, to = y_max_ext, length.out =
n_ext)
} else {
```

```
  extreme_points$y_special <- numeric(0)
}

# —— 先找绝对估计值最大的前 10 个下标 ——
top_10_indices <- order(-abs(data1$Estimate))[1:10]
top_10_meta      <- data1$Metabolite[top_10_indices]
# —— 构建 significant_points 时，把 logp 分两种情况 ——
significant_points <- data.frame(
  estimate = data1$Estimate[top_10_indices],
  logp       = sapply(top_10_meta, function(m) {
    # 如果原来的 -log10(p) = Inf，就匹配 extreme_points 中的 y_special
    orig_logp <- data1$logp[ data1$Metabolite == m ]
    if (is.infinite(orig_logp)) {
      # 匹配到 extreme_points 那一行，拿 y_special
      return(extreme_points$y_special[ match(m, extreme_points$Metabolite) ])
    } else {
      # 否则就用原本的有限值（或者用 logp_capped）
      return(orig_logp)
    }
  }),
  metabolic = top_10_meta,
  stringsAsFactors = FALSE
)

y_tick_max <- floor(cap)
# —— 5. 开始绘图 ——
gg <- ggplot() +

  # 5.1 先画灰色半透明区块——区块范围 x 先定极限，y 从 cap 到 cap*1.3
  geom_rect(aes(xmin = -Inf, xmax = Inf,
                    ymin = cap,          # 灰色区块下界
                    ymax = cap * 1.3), # 灰色区块上界
                fill = "grey80", alpha = 0.5) +
  geom_hline(yintercept = -log10(0.05), linetype = 2, color = "black", size = 1) +

  # 5.2 绘制“普通”点：y 用 logp_capped，x 用 Estimate
  geom_point(data = normal_points,
               aes(x = Estimate, y = logp_capped, color = color),
               size = 3) +

  # 5.3 绘制“被截断”的点：用 y_special 作为纵坐标
  geom_point(data = extreme_points,
               aes(x = Estimate, y = y_special, color = color),
               size = 3) +
```

```
# 5.4 高亮前 10 个显著点（如果属于截断，用 y_special；否则用 logp_capped）
geom_point(data = significant_points,
           aes(x = estimate, y = logp),
           color = "gold", size = 4) +

# 5.5 在灰色区块下方画一条虚线，提示“所有 p 极小，集中展示”


# 5.6 给前 10 个显著点加标签
geom_text_repel(data = significant_points,
                aes(x = estimate, y = logp, label = metabolic),
                size = 5,                          # 调大标签字体
                color = "black",
                box.padding     = unit(0.4, "lines"),
                segment.color = "black",
                segment.size    = 0.6,
                min.segment.length = 0.5) +

# 5.7 美化：自定义配色、去网格、统一字体黑色并增大
scale_color_manual(values = c(
  'positive' = '#DE3024',
  'negative' = '#25108f',
  'no'           = 'grey'
)) +

# ** 关键：y 轴上限直接设为 cap * 1.3，与灰色区块 top 对齐 **
scale_y_continuous(
  limits = c(0, cap * 1.3),   # 下限可以从 0 或最小 logp 开始
  expand = c(0, 0) ,
  breaks = seq(0, y_tick_max, by = 4)# 去掉额外留白
) +

# 5.8 主题设置：所有文字黑色，字体统一变大
theme(
  plot.margin = margin(),
  panel.background     = element_rect(fill = 'transparent', color = 'transparent'),
  panel.grid.major     = element_blank(),
  panel.grid.minor     = element_blank(),
  axis.line.x            = element_line(size = 0.7, color = "black"),
  axis.line.y            = element_line(size = 0.7, color = "black"),
  axis.text               = element_text(size = 14, color = "black"),     # 刻度文字
  axis.title             = element_text(size = 16, color = "black"),     # 坐标轴标题
  axis.ticks              = element_line(size = 0.7, color = "black"),
```

```
    legend.position       = "none",
    plot.title              = element_text(size = 18, color = "black", face = "bold")
  ) +

  # 5.9 坐标轴及标题
  labs(
    x = "Coefficient",
    y = "-log10(p value)",
    title = ""     # 如果需要标题，可以在这里填写；否则保持空
  )+
  coord_trans(y = squash_axis(0, cap, 3))

gg
ggsave("CH_geeglm_meta.pdf", width = 5, height = 6)

####plot DH####
data1<-read.csv('DH_GEEglm_meta.csv',row.names = 1)
data1$logp            <- -log10(data1$p_value)
finite_vals        <- data1$logp[is.finite(data1$logp)]
max_finite          <- if (length(finite_vals)>0) max(finite_vals, na.rm=TRUE) else 0
cap                     <- max_finite + 1   # 设定灰色区块的上界
data1$logp_capped <- ifelse(is.infinite(data1$logp), cap, data1$logp)
data1$color<- "no"
data1$color[(data1$p_value<0.05)&(data1$Estimate>0)] <- "positive"
data1$color[(data1$p_value<0.05)&(data1$Estimate<0)]   <- "negative"
table(data1$color)
# —— 2. 找到所有被截断的点（p 极小）和普通点 ——
extreme_points <- subset(data1, is.infinite(logp))      # 原始 logp 为 Inf 的那些行
normal_points   <- subset(data1, is.finite(logp))         # logp 有限的行

# —— 3. 给被截断点设计一个“美观的 y 坐标” ——
#       根据 Estimate 的绝对值排序，然后在区块内均匀分配 y 值
extreme_points <- extreme_points[order(abs(extreme_points$Estimate)), ]

n_ext <- nrow(extreme_points)
if(n_ext > 0) {
  # 目标区间 [cap * 0.8, cap * 0.98]，你可以根据需要调节
  y_min_ext <- cap*1.01
  y_max_ext <- cap *1.28

  # 在这个范围内等距分配
  extreme_points$y_special <- seq(from = y_min_ext, to = y_max_ext, length.out =
n_ext)
} else {
```

```
  extreme_points$y_special <- numeric(0)
}

# —— 先找绝对估计值最大的前 10 个下标 ——
top_10_indices <- order(-abs(data1$Estimate))[1:10]
top_10_meta      <- data1$Metabolite[top_10_indices]
# —— 构建 significant_points 时，把 logp 分两种情况 ——
significant_points <- data.frame(
  estimate = data1$Estimate[top_10_indices],
  logp       = sapply(top_10_meta, function(m) {
    # 如果原来的 -log10(p) = Inf，就匹配 extreme_points 中的 y_special
    orig_logp <- data1$logp[ data1$Metabolite == m ]
    if (is.infinite(orig_logp)) {
      # 匹配到 extreme_points 那一行，拿 y_special
      return(extreme_points$y_special[ match(m, extreme_points$Metabolite) ])
    } else {
      # 否则就用原本的有限值（或者用 logp_capped）
      return(orig_logp)
    }
  }),
  metabolic = top_10_meta,
  stringsAsFactors = FALSE
)

y_tick_max <- floor(cap)
# —— 5. 开始绘图 ——

gg <- ggplot() +

  # 5.1 先画灰色半透明区块——区块范围 x 先定极限，y 从 cap 到 cap*1.3
  geom_rect(aes(xmin = -Inf, xmax = Inf,
                ymin = cap,         # 灰色区块下界
                ymax = cap * 1.3), # 灰色区块上界
            fill = "grey80", alpha = 0.5) +
  geom_hline(yintercept = -log10(0.05), linetype = 2, color = "black", size = 1) +

  # 5.2 绘制“普通”点：y 用 logp_capped，x 用 Estimate
  geom_point(data = normal_points,
             aes(x = Estimate, y = logp_capped, color = color),
             size = 3) +

  # 5.3 绘制“被截断”的点：用 y_special 作为纵坐标
  geom_point(data = extreme_points,
             aes(x = Estimate, y = y_special, color = color),
```

```
                     size = 3) +

# 5.4 高亮前 10 个显著点（如果属于截断，用 y_special；否则用 logp_capped）
geom_point(data = significant_points,
           aes(x = estimate, y = logp),
           color = "gold", size = 4) +

# 5.5 在灰色区块下方画一条虚线，提示“所有 p 极小，集中展示”


# 5.6 给前 10 个显著点加标签
geom_text_repel(data = significant_points,
                aes(x = estimate, y = logp, label = metabolic),
                size = 5,                    # 调大标签字体
                color = "black",
                box.padding     = unit(0.4, "lines"),
                segment.color = "black",
                segment.size   = 0.6,
                min.segment.length = 0.5) +

# 5.7 美化：自定义配色、去网格、统一字体黑色并增大
scale_color_manual(values = c(
  'positive' = '#DE3024',
  'negative' = '#25108f',
  'no'          = 'grey'
)) +

# ** 关键：y 轴上限直接设为 cap * 1.3，与灰色区块 top 对齐 **
scale_y_continuous(
  limits = c(0, cap * 1.3),   # 下限可以从 0 或最小 logp 开始
  expand = c(0, 0) ,
  breaks = seq(0, y_tick_max, by = 4)# 去掉额外留白
) +

# 5.8 主题设置：所有文字黑色，字体统一变大
theme(
  plot.margin = margin(),
  panel.background     = element_rect(fill = 'transparent', color = 'transparent'),
  panel.grid.major     = element_blank(),
  panel.grid.minor     = element_blank(),
  axis.line.x            = element_line(size = 0.7, color = "black"),
  axis.line.y            = element_line(size = 0.7, color = "black"),
  axis.text               = element_text(size = 14, color = "black"),     # 刻度文字
  axis.title             = element_text(size = 16, color = "black"),     # 坐标轴标题
```

```
    axis.ticks                = element_line(size = 0.7, color = "black"),
    legend.position        = "none",
    plot.title              = element_text(size = 18, color = "black", face = "bold")
  ) +

  # 5.9 坐标轴及标题
  labs(
    x = "Coefficient",
    y = "-log10(p value)",
    title = ""      # 如果需要标题，可以在这里填写；否则保持空
  )+
  coord_trans(y = squash_axis(0, cap, 3))

gg
ggsave("DH_geeglm_meta.pdf", width = 5, height = 6)
```
